# Supplementary material for: Structural insights into RNA encapsidation and helical assembly of the Toscana virus nucleoprotein
Source: Nucleic Acids Res. 2014 Mar 31;42(9):6025–37. doi: 10.1093/nar/gku229 (PMC4027202; doi:10.1093/nar/gku229)
Supplement: SUPPLEMENTARY DATA [file supp_42_9_6025__index.html]

Structural insights into RNA encapsidation and helical assembly of the Toscana virus nucleoprotein — Structural insights into RNA encapsidation and helical assembly of the Toscana virus nucleoprotein — SUPPLEMENTARY DATA 

# Structural insights into RNA encapsidation and helical assembly of the Toscana virus nucleoprotein

## SUPPLEMENTARY DATA

**Files in this Data Supplement:**

- SUPPLEMENTARY DATA
