# Supplementary material for: Structural insights into RNA encapsidation and helical assembly of the Toscana virus nucleoprotein
Source: Nucleic Acids Res. 2014 Mar 31;42(9):6025–37. doi: 10.1093/nar/gku229 (PMC4027202; doi:10.1093/nar/gku229)
Supplement: SUPPLEMENTARY DATA [file supp_gku229_nar-02240-r-2013.zip › nar-02240-r-2013-File007.pdf]

Supplementary Material

Article: Structural insights into RNA encapsidation and helical assembly of the Toscana virus nucleoprotein

Authors: Daniel Olal, Alexej Dick, Virgil L. Woods Jr, Tong Liu, Sheng Li, Stephanie Devignot, Friedemann Weber, Erica Ollmann Saphire and Oliver Daumke

Supplementary Figure S1: **Initial characterization of N**

Supplementary Figure S2: **Binding of N to 9mer RNA sequences**

Supplementary Figure S3: **Contents of the asymmetric units**

Supplementary Figure S4: **Sequence alignment**

Supplementary Figure S5: **Interactions and dynamics of the amino-terminal arm**

Supplementary Figure S6: **Structural comparison of Toscana virus N subunits**

Supplementary Figure S7: **Modeling the binding of oligo3**

Supplementary Figure S8: **Inter-subunit rearrangements in Toscana Virus RNPs**

Supplementary Figure S9: **Inter-subunit rearrangements in RVFV RNPs**

Supplementary Figure S10: **Comparison with tenuiviruses N**

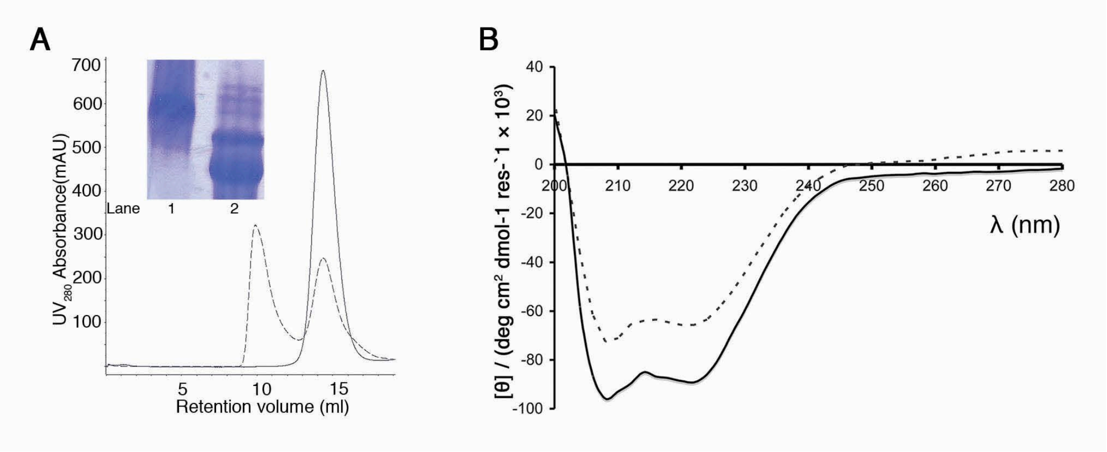

Supplementary Figure S1: Initial characterization of N

(**A**) Elution profile of the refolded N (dashed line) and the reconstituted RNP (solid line). The inset shows an electromobility shift assay with (1) refolded N and (2) RNP reconstituted with nonameric RNA. The RNP complex migrates faster due to the additional negative charge conferred by the bound nonameric RNA.

(**B**) Circular dichroism spectra of Toscana virus N with (solid line) and without (dashed line) an unfolding/refolding step. The molar ellipticity θ was measured at room temperature between 200 and 290 nm. The similar profiles suggest that the refolding step did not grossly affect the structure of N.

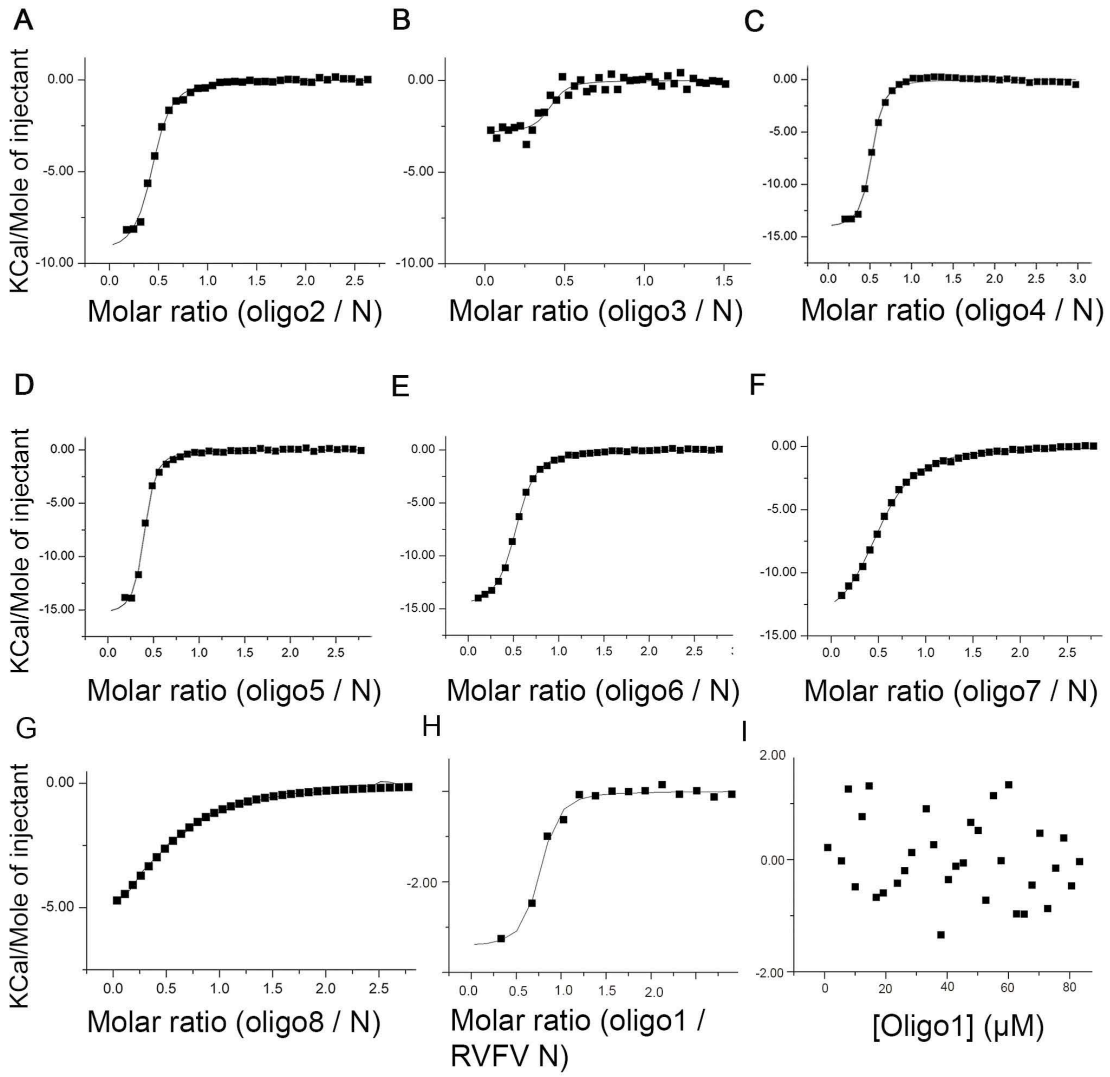

Supplementary Figure S2: Binding of N to 9mer RNA sequences

ITC experiments were performed as described in Fig. 1b. Shown are binding curves of Toscana virus N to (A) oligo2, a 9mer RNA corresponding to the 3' genomic consensus sequence UCUUUGUGU (K_d = 560 nM ± 70 nM, n = 0.43 ± 0.01). (B) oligo3, a 9mer RNA corresponding to the 5' genomic consensus ACACAGAGA. The binding signal of latter experiment was too weak for a reliable fitting of the binding parameters. This does not rule out that the binding affinity is in a similar range as for the other 9mer RNAs. (C) oligo4, a 9mer RNA with the sequence UAUGUUUCU (K_d = 290 nM ± 40 nM, n = 0.49 ± 0.01). (D) oligo5, a polyU 9mer RNA (K_d = 360 nM ± 40 nM, n = 0.40 ± 0.01). (E) oligo6 comprising the 8mer UGUGUUUC (K_d = 710 nM ± 30 nM, n= 0.51 ± 0.01). (F) oligo7 comprising the 7mer UGUGUUU (K_d = 2.7 µM ± 0.2 µM, n= 0.52 ± 0.01). (G) oligo8 comprising the 6mer UGUGUU (K_d = 8.0 µM ± 0.9 µM, n= 0.50 ± 0.03). (H) RVFV N to oligo1 (K_d = 400 nM ± 100 nM, n = 0.70± 0.02) (I) Control injection of oligo1 UGUGUUUCU into buffer.

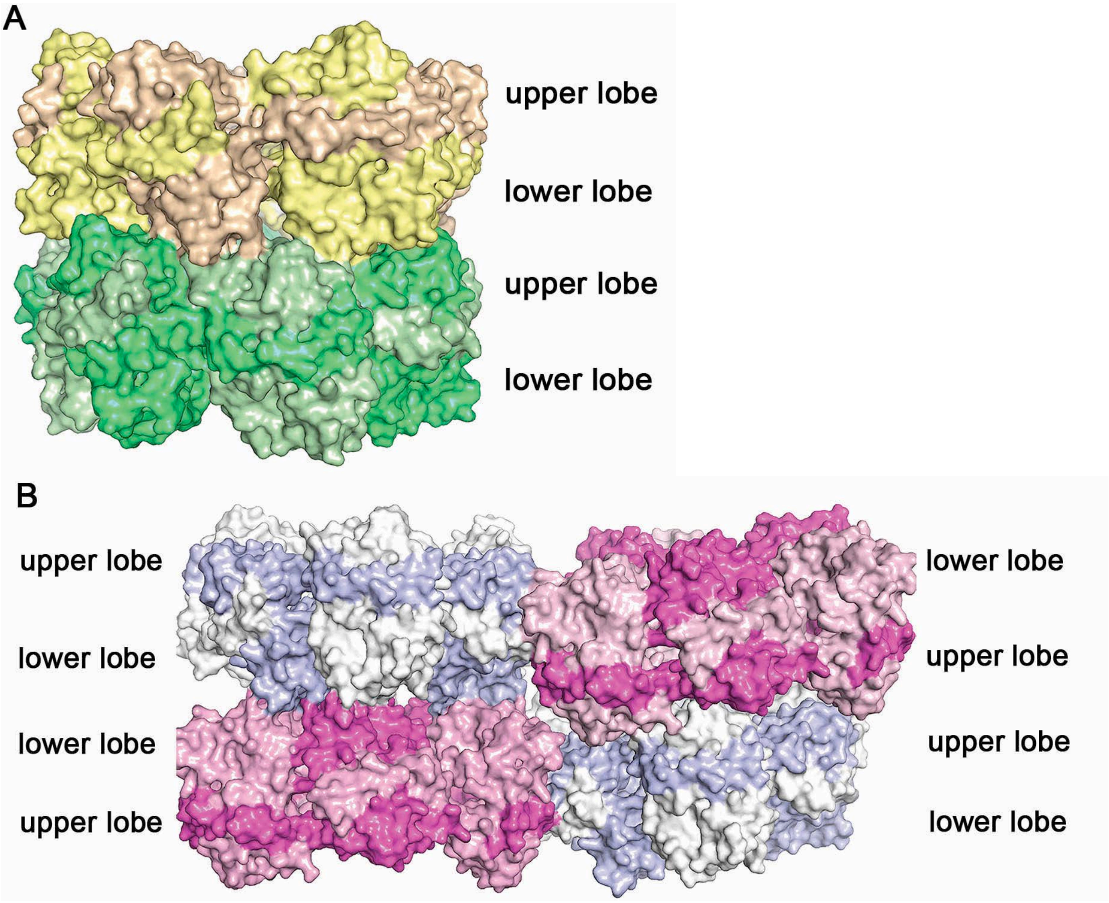

Supplementary Figure S3. Contents of the asymmetric units
(**A**) The apo structure contained two hexameric rings in the asymmetric unit that were stacked on top of each other. (**B**) In the RNP structure, four hexameric rings were assembled in a head-to-head fashion in the asymmetric unit.

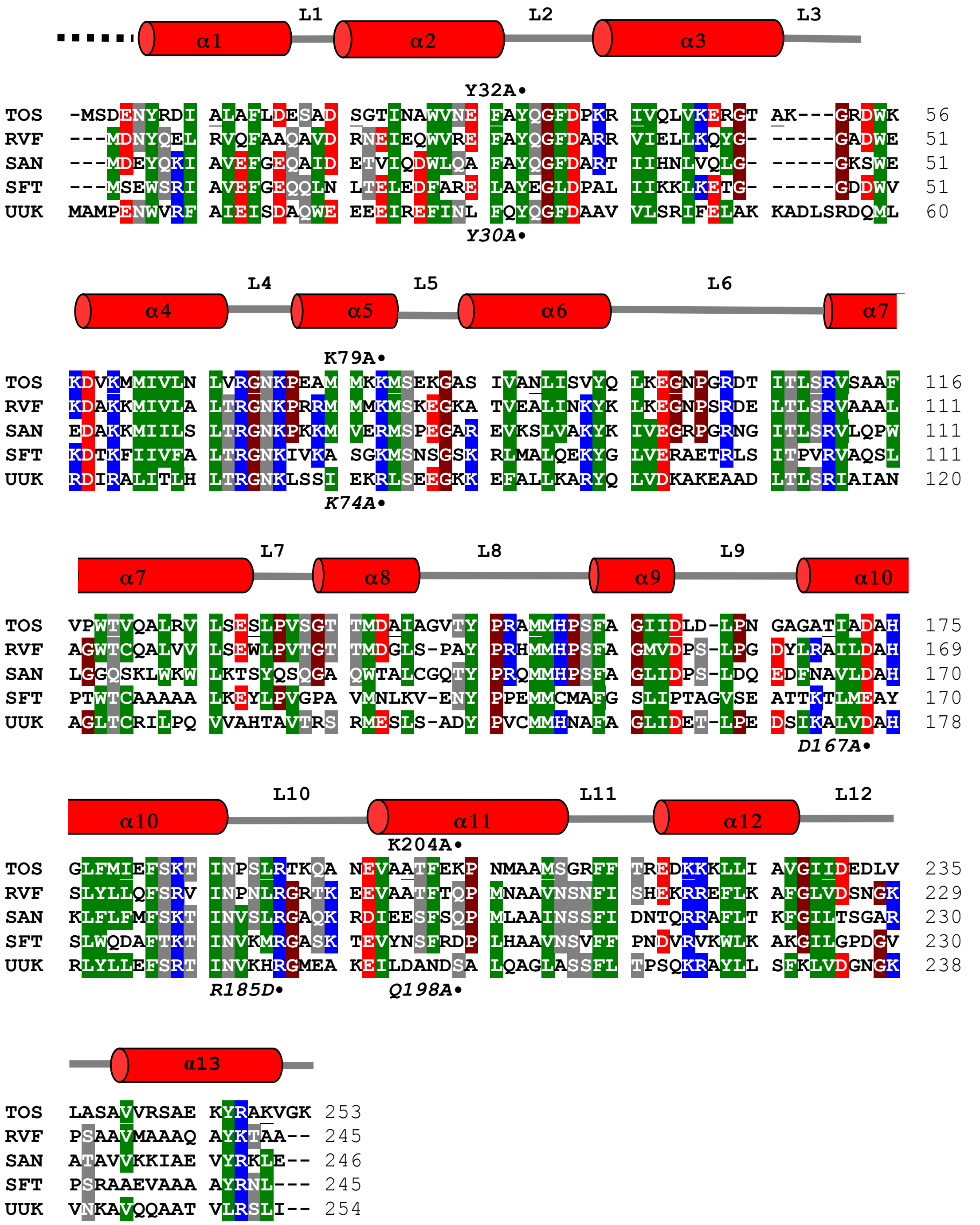

Supplementary Figure S4: Sequence alignment

The following sequences from different phlebovirus species were aligned (Swissprot accession codes are shown in brackets): **To**scana virus (P21701), **R**ift **V**alley **F**ever virus (P21700), **San**dfly Fever Sicilian virus (P12793), **S**evere **F**ever with **T**hrombocytopenia Syndrome (SFTS) virus (F1BWV8) and **Uuk**uniemi virus (P22025). Residues with a sequence conservation of 60% and greater are color-coded (D,E in red; R,K,H in blue; N,Q,S,T in grey; L, I, V, F, Y, W, M, C in green, P,G in brown). Chain A of the RNA-free N was used as a reference to determine the secondary structure, shown on top of the alignment, with alpha-helices indicated as cylinders. Mutations introduced in Toscana virus N are shown above and in RVFV N below the alignment.

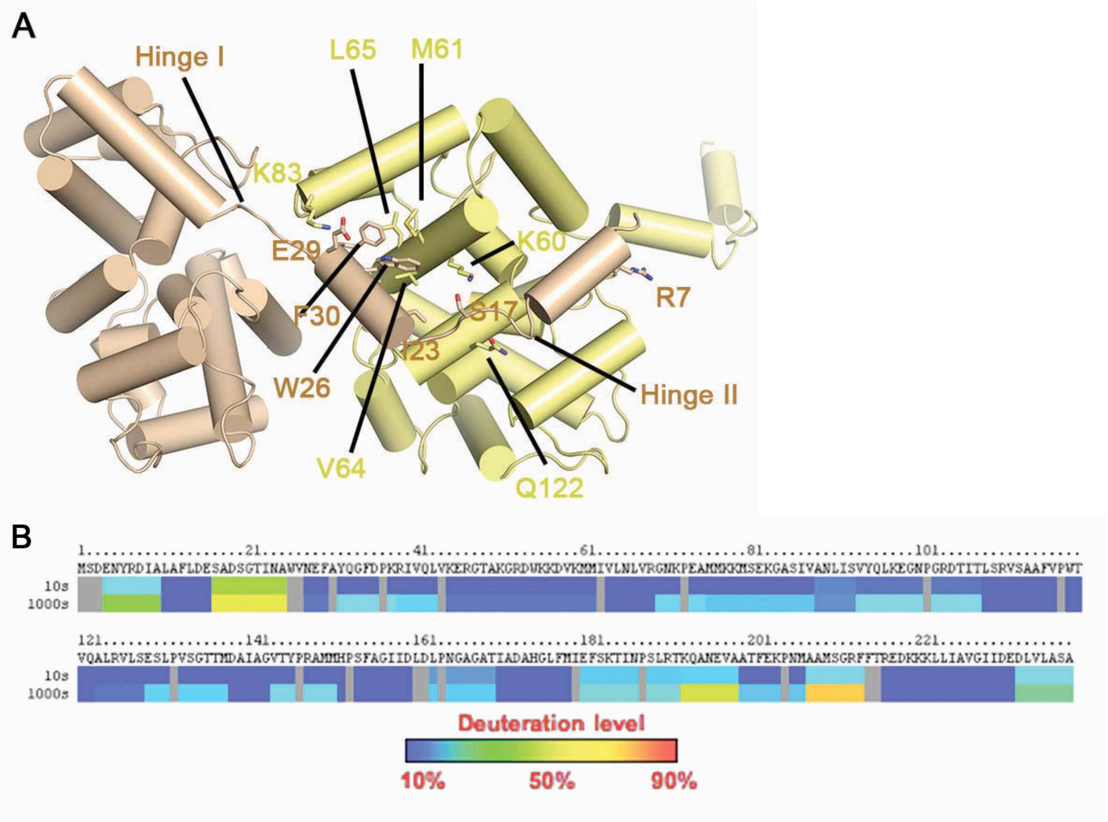

Supplementary Figure S5. Interactions and dynamics of the amino-terminal arm
(A) Two adjacent N monomers from the apo structure illustrate the inter-subunit interactions mediated by the amino-terminal arm.
(B) Deuterium exchange mass spectrometry shows that hinge II in the amino-terminal arm undergoes rapid hydrogen exchange pointing to a high degree of flexibility.

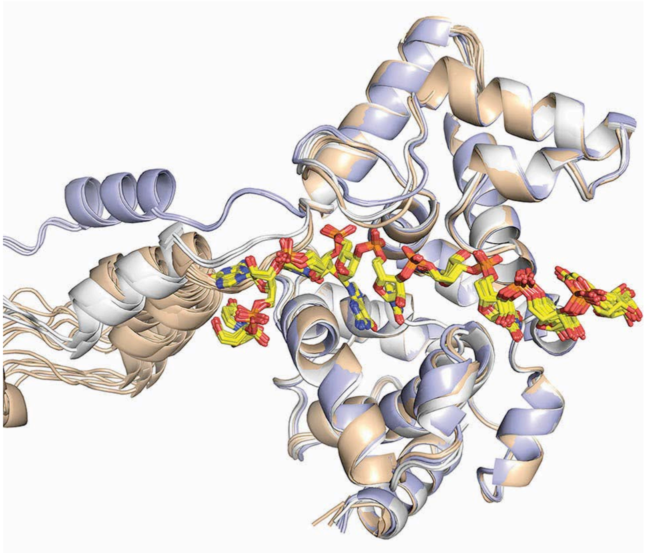

Supplementary Figure S6: Structural comparison of Toscana virus N subunits
Superposition of the Toscana virus N and RNP subunits from our structures (brown: 12 apo Ns, white: 12 Ns without bound RNA from the RNP, blue: 12 9mer RNA-bound subunits). The structural alignment reveals that the amino-terminal arm of all RNA-liganded subunits is displaced relative to their unliganded counterparts.

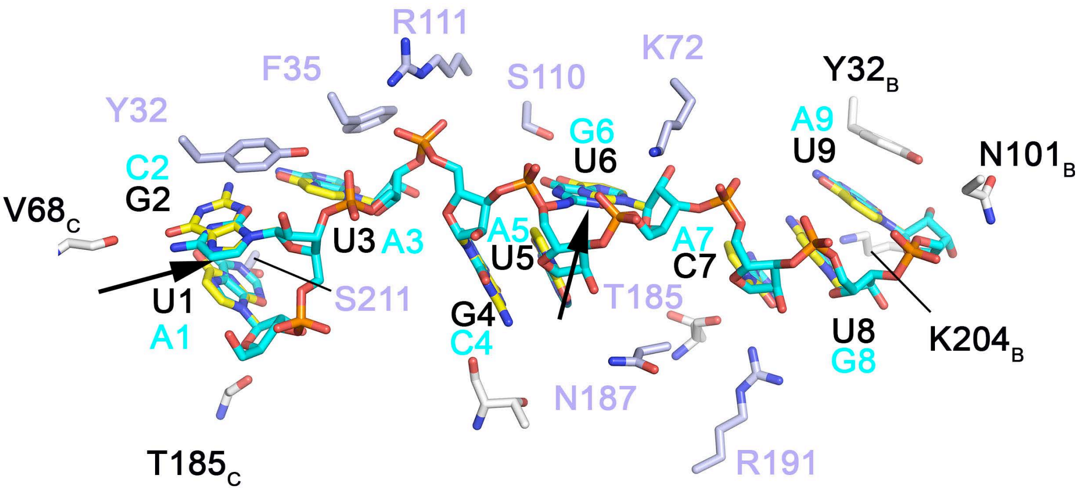

Supplementary Figure S7: Modeling the binding of oligo3

Assuming an identical backbone conformation as observed for oligo1 (UGUGUUUCU, yellow) in the RNP structures, steric clashes (indicated as arrows) are predicted to occur when oligo3 (ACACAGAGA, cyan) binds into the RNA binding groove, e.g. between nucleotides 1 and 2 and nucleotides 5 and 6. This may account for the diminished binding affinity of oligo3 to the N protein.

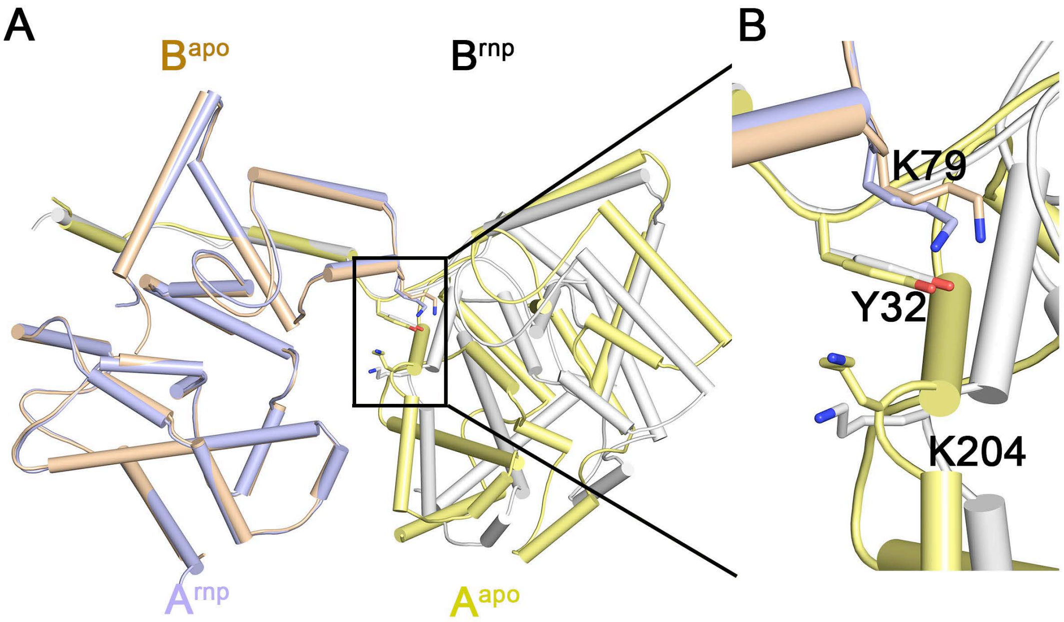

Supplementary Figure S8: Inter-subunit rearrangements in Toscana virus RNPs
(A) Superposition of two N monomers at the 3' RNA end of the RNP (purple and white) with two monomers from the apo structure (orange and yellow), similarly as in Fig. 4c where two RNP subunits at the 5' RNA end were superimposed with the apo structure. The box in (A) is magnified in (B). The analysis reveals that Tyr32, Lys79 and Lys204 in the apoprotein and in the RNP at the 3' RNA junction occupy similar positions. Consequently, the neighboring N is not much rotated compared to the junction at the 5' RNA end in the RNP.

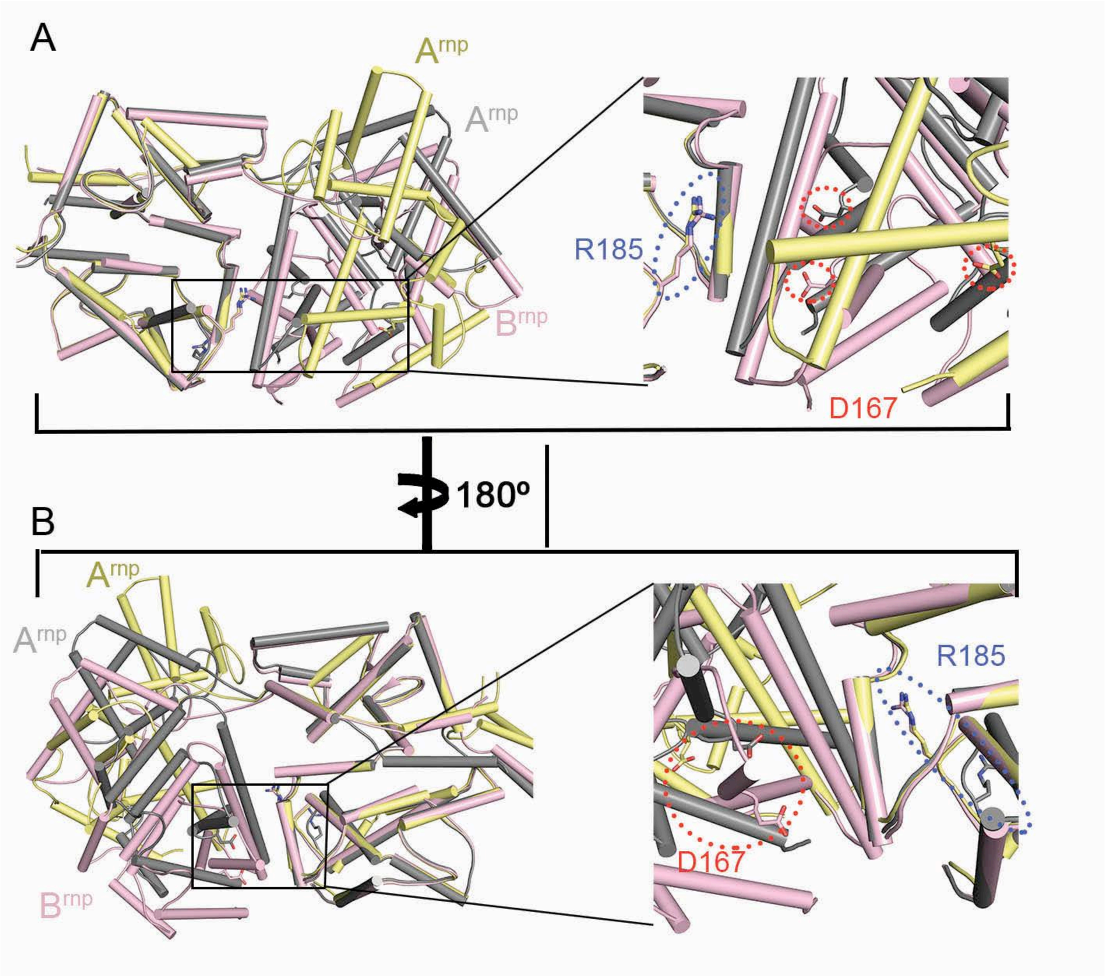

Supplementary Figure S9: Inter-subunit rearrangements in RVFV RNPs

(A) Superposition of two molecules of the RVFV RNP tetramers bound to single stranded RNA (PDB codes: 4H5P, yellow), of the RVFV N hexamers bound to single stranded DNA (4H5Q, grey) and of the RVFV RNP hexamer bound to single stranded RNA (4H6F, magenta). A magnification of the black box is shown at the right. The comparison reveals that RNA binding does not lead to the formation of a salt bridge between Asp167 and Arg185 (Asp173 and Arg191 in Toscana virus N) and, consequently, no additional dimer interface is formed. For clarity, the RNA is not shown. Compare with Fig. 4f.

(B) View on the superimposed RNPs structures from the outside of the hexamer. A magnification of the box is shown at the right. The inter-subunit contact is exclusively mediated by the amino terminal arm and no additional interface is formed.

A

```
RHB   MTMSVADVQT EIERVTTLAL KYITDHKDTL VT--FVGQIE YNGY-DAGKL LQILKRKAE-  56
TOS   ---------- --ENYRDI L AFLDESADSG TINAWVNEFA YQGF-DPKRI VQLVKERGTA  50
RVF   ---------- --DNYQELAI QFAAQAVDNE IEQ-WVREFA YQGF-DARRV IELLKQYG--  46

RHB   --GRDFGKDL CYLLVMRYTR GTGFVRDVRK KIKTAAGGDT AHEIVTHYGV VQSVGDNADA  114
TOS   -KGRDWKKDV KMMIVLNLVR GNKPEAMMKK --MSEKGASI VANLISVYQL KEG-NPGRDT  106
RVF   --GADWEKDA KKMIVLALTR GNKPRRMMMK --MSKEGKAT VEALINKYKL KEG-NPSRDE  101

RHB   ITLGRLASLF PAVSMNIVRN VSTGAKLAVD SSDLGSSGLD LLLWDFVPQF ITLDSLTAPY  174
TOS   ITLSRVSAAF VPWTVQALRV LS-ESLPVSG TTMDAIAGVT YPRAMMHPSF AGIIDLDLPN  165
RVF   LTLSRVAAAL AGRTCQALVV LS-EWLPVTG TTMDGLSPAY PRHMMH-PSF AGMVDPSLPG  159

RHB   CTGKGM--NT ILFSLHVLHG ALTTKKTMPD QKKKARSLVT DFELVKYTTE LLVITCNAKN  232
TOS   GAGATIADAH GLFMIEFSKT INPSLRTKQ- --ANEVAATF EKPNMAAMS- ----------  211
RVF   DYLRAILDAH SLYLLQFSRV INPNLRGRT- --KEEVAATF TQPMNAAVN- ----------  205

RHB   LKVEKKTIYR ESLMRPFQEG AGDALGFKET FWTSLAKVST GCVRKMKKDA QAYLSERTPL  292

RHB   LKVFVENCNK SEDEVAEAIK SYFVTGA 319
```

B

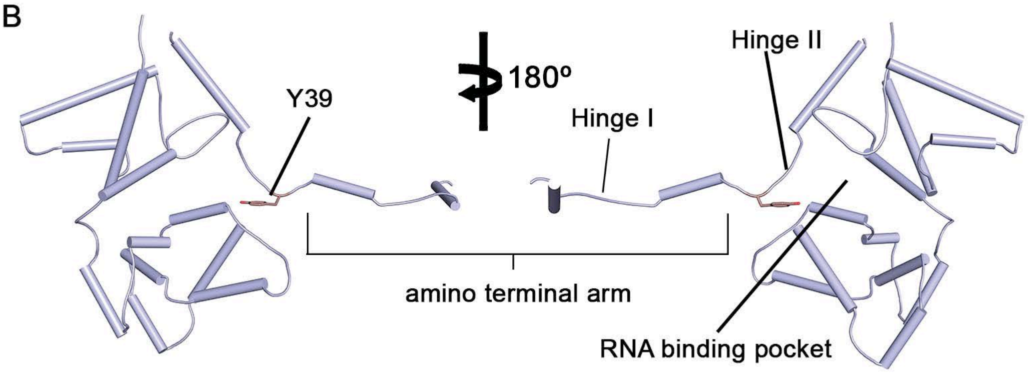

C

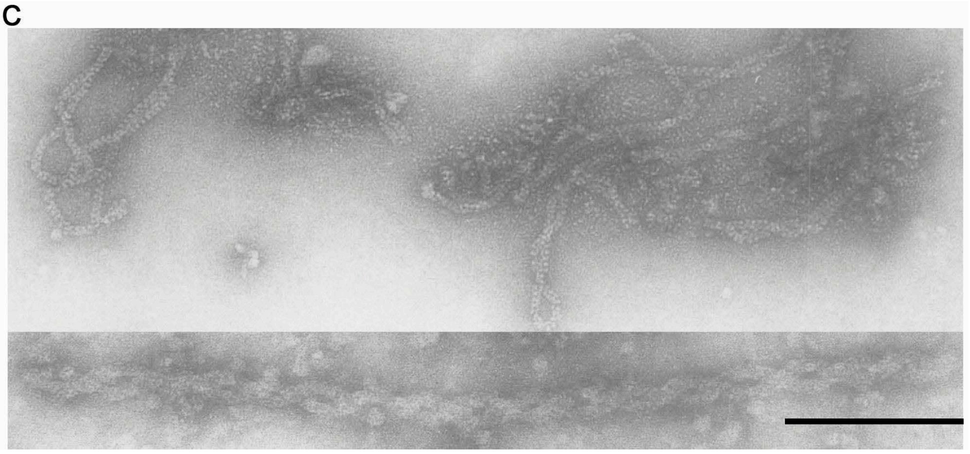

Supplementary Figure S10: Comparison with tenuiviruses N

(A) Sequence alignment of the N of Toscana virus (TOS) and Rift Valley Fever virus (RVF) with Rice Hoja Blanca (RHB) tenuivirus (Swiss prot accession code Q67898). This alignment was used for the structure prediction in (B).

(B) Structural prediction of the N of Rice Hoja Blanca tenuivirus using the homology server phyre (44), from front and back view. 61% of the sequence was modeled with 100% confidence.

(C) EM micrographs of tenuiviruses (taken from reference 44). The upper picture shows the filamentous organization of the RNP, with a distinct helical sub-pattern. The second picture shows super-coiling of the RNP filaments. Scale bar is 100 nm.
