# Supplementary figures and images for: Structural insights into RNA encapsidation and helical assembly of the Toscana virus nucleoprotein
Source: Nucleic Acids Res. 2014 Mar 31;42(9):6025–37. doi: 10.1093/nar/gku229 (PMC4027202; doi:10.1093/nar/gku229)

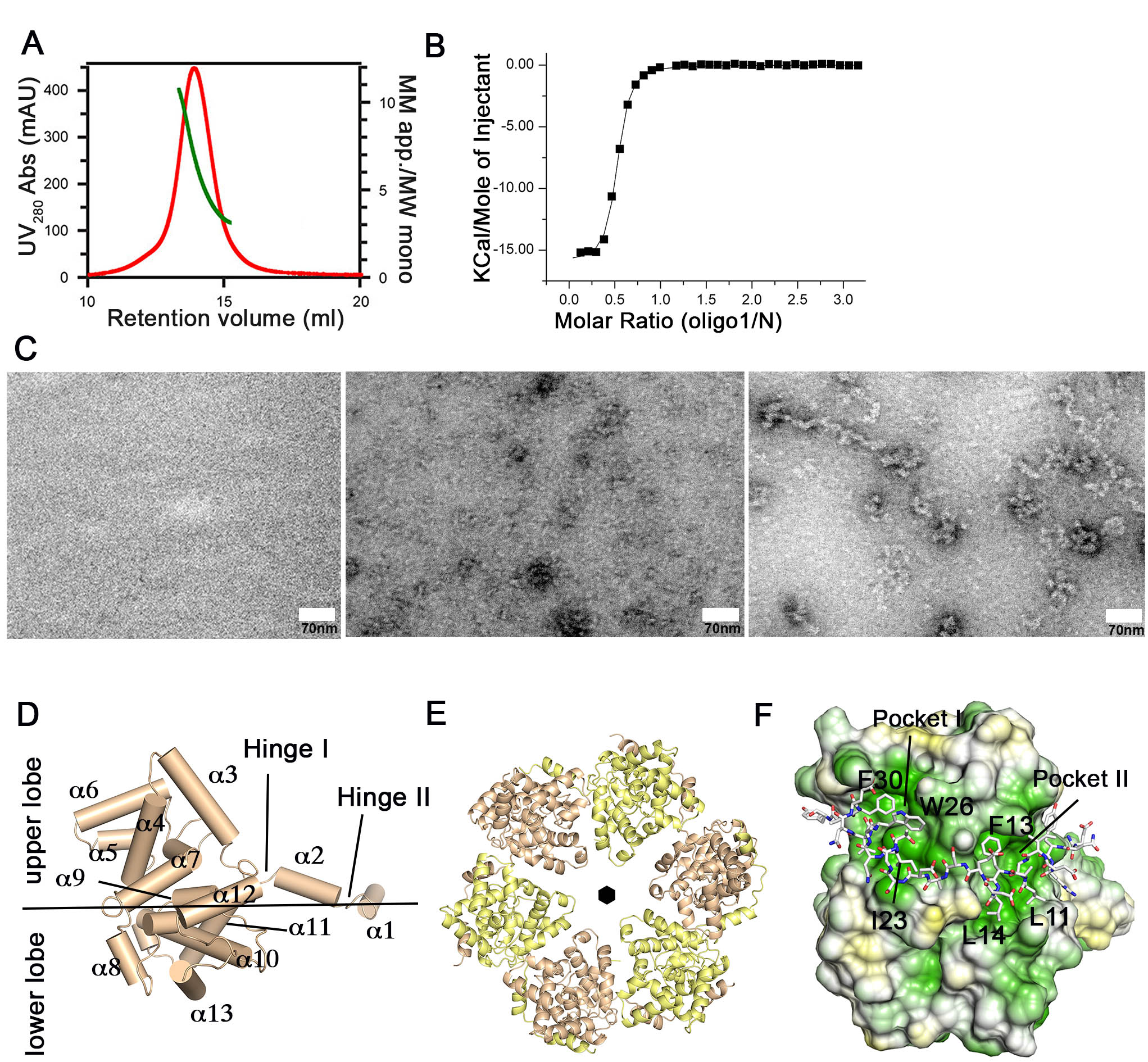

Supplement: SUPPLEMENTARY DATA [file supp_gku229_nar-02240-r-2013.zip › nar-02240-r-2013-File002.jpg]

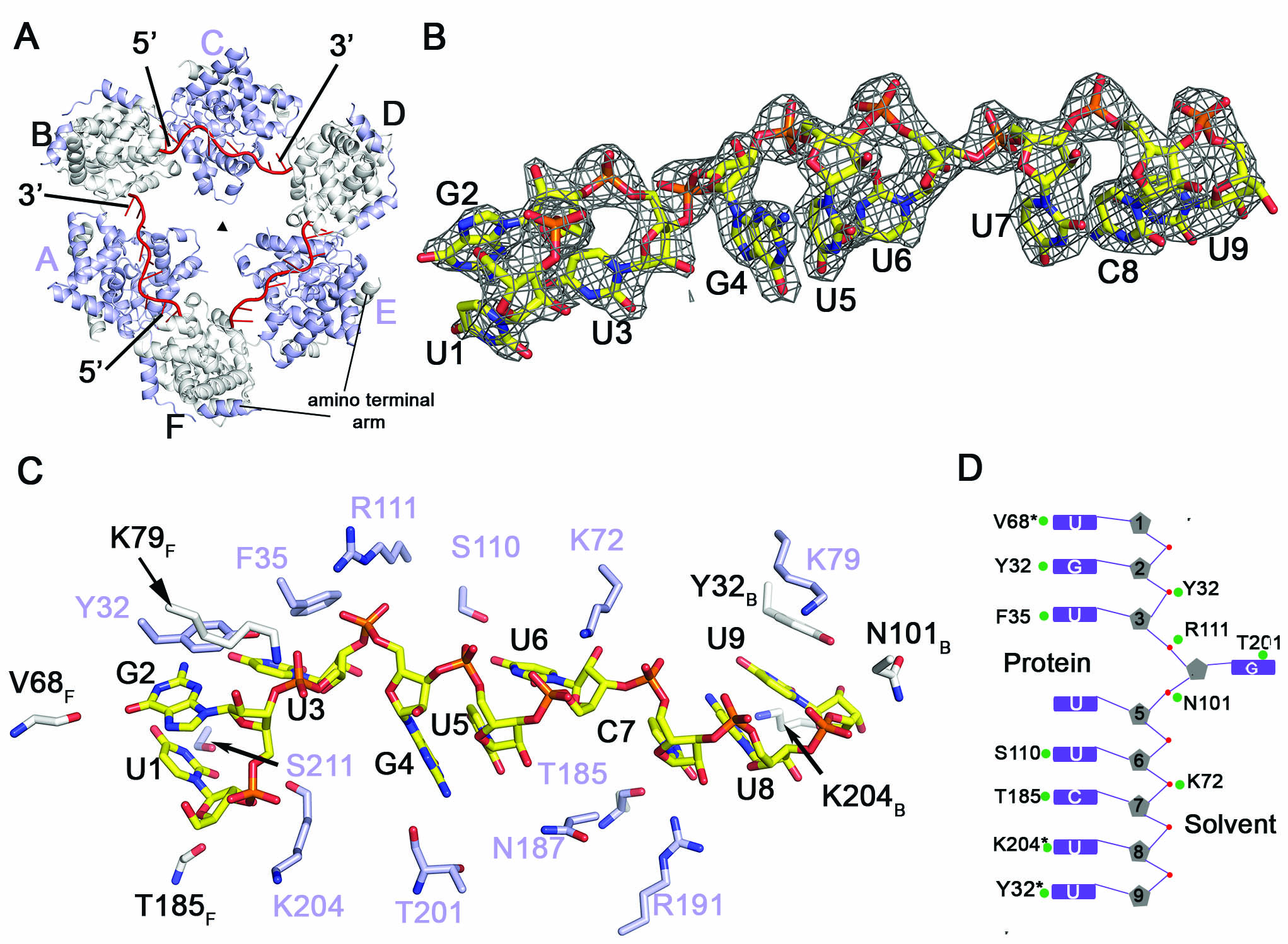

Supplement: SUPPLEMENTARY DATA [file supp_gku229_nar-02240-r-2013.zip › nar-02240-r-2013-File003.jpg]

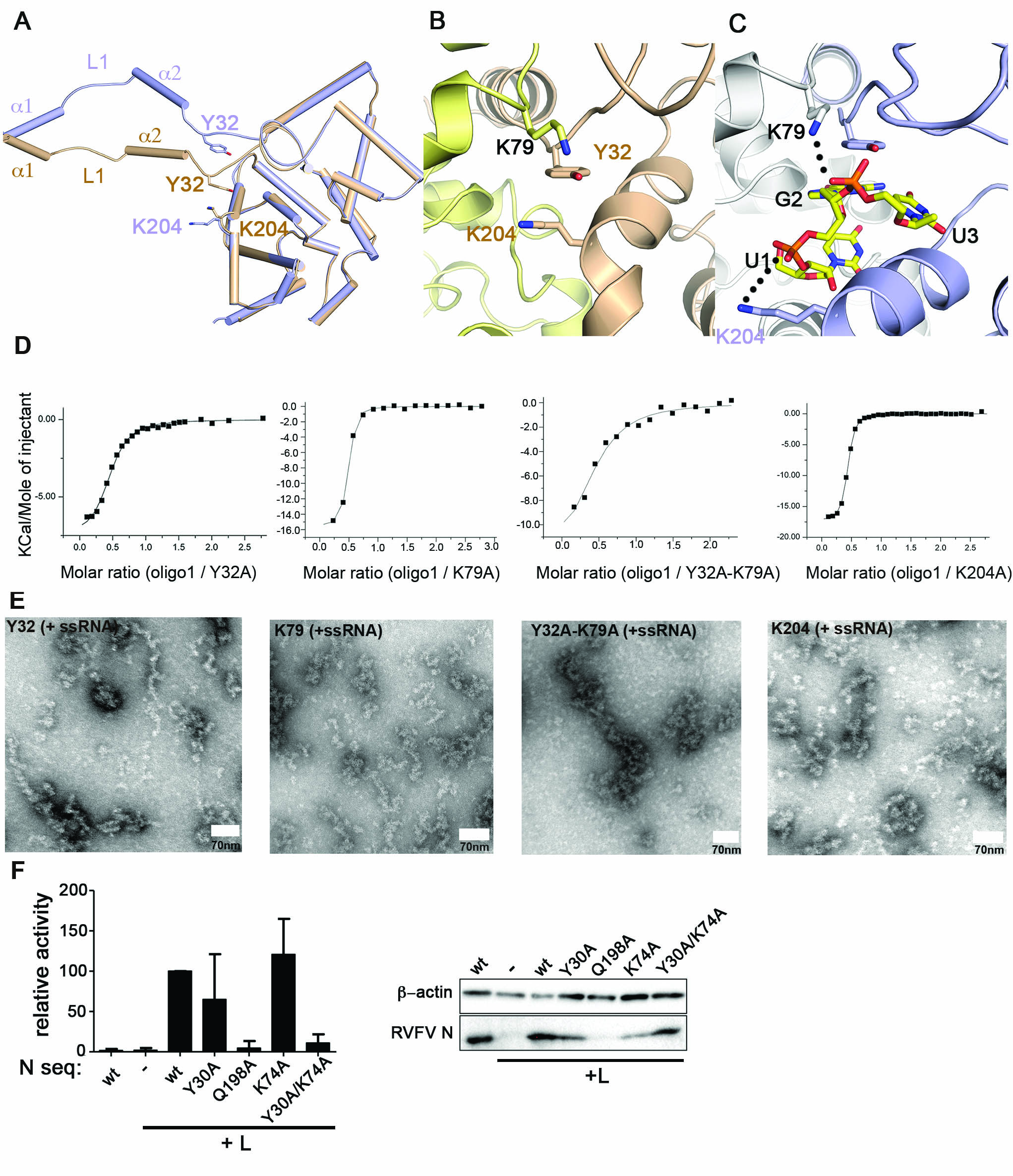

Supplement: SUPPLEMENTARY DATA [file supp_gku229_nar-02240-r-2013.zip › nar-02240-r-2013-File004.jpg]

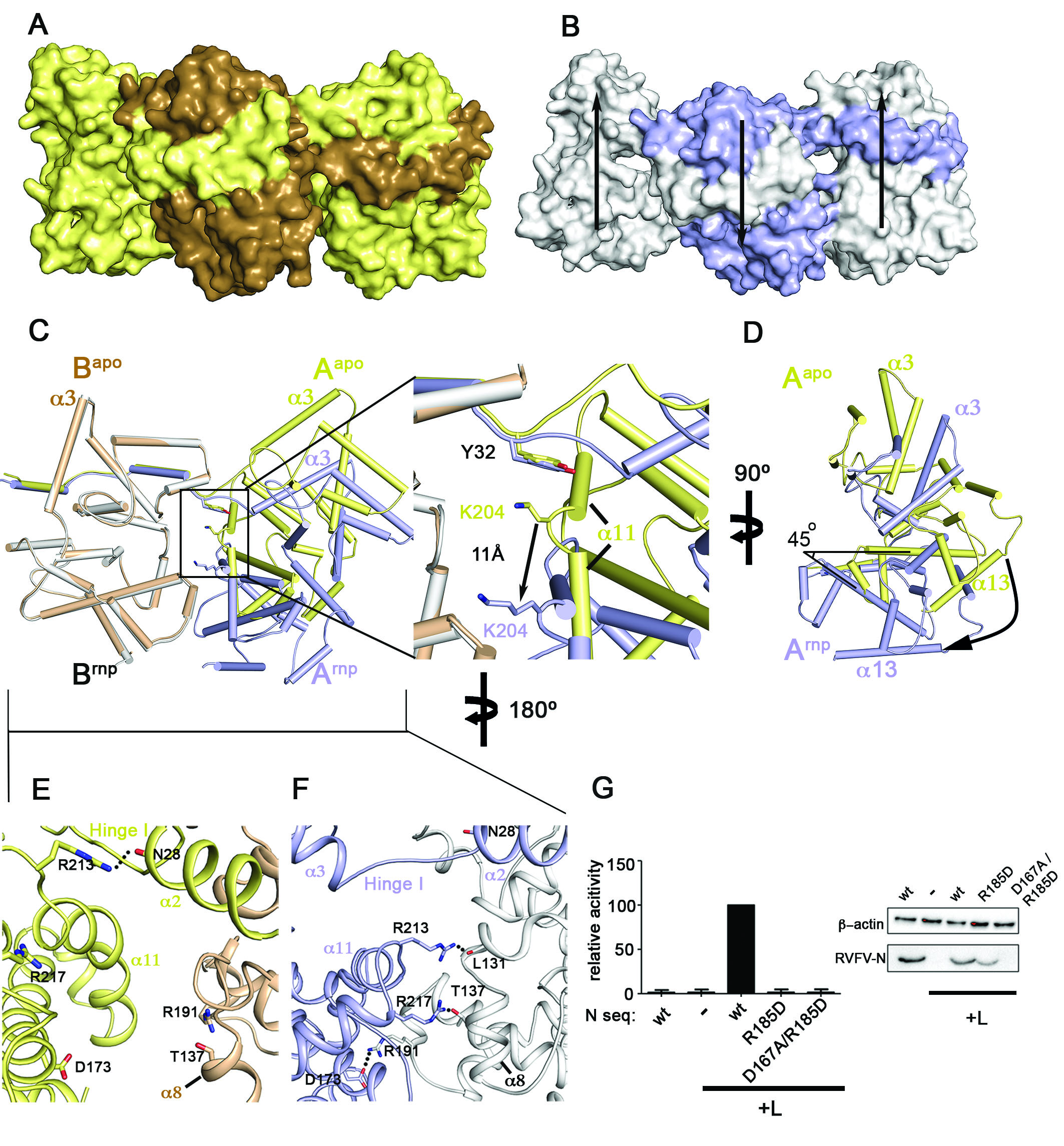

Supplement: SUPPLEMENTARY DATA [file supp_gku229_nar-02240-r-2013.zip › nar-02240-r-2013-File005.jpg]

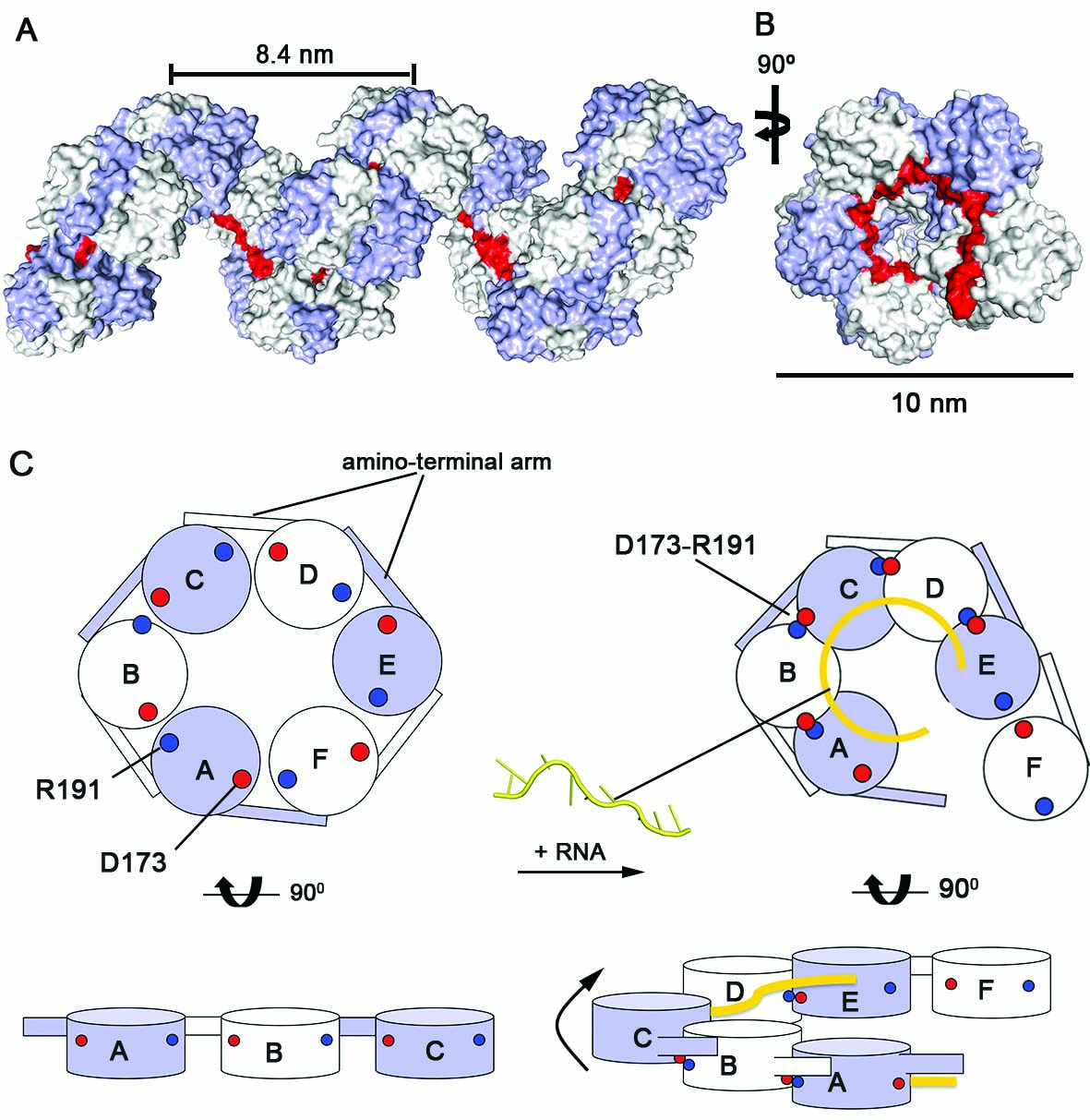

Supplement: SUPPLEMENTARY DATA [file supp_gku229_nar-02240-r-2013.zip › nar-02240-r-2013-File006.jpg]
