# Supplementary material for: Structural insights into RNA encapsidation and helical assembly of the Toscana virus nucleoprotein
Source: Nucleic Acids Res. 2014 Mar 31;42(9):6025–37. doi: 10.1093/nar/gku229 (PMC4027202; doi:10.1093/nar/gku229)
Supplement: SUPPLEMENTARY DATA [file supp_gku229_nar-02240-r-2013.zip › nar-02240-r-2013-File001.doc]

**Structural insights into RNA encapsidation and helical assembly of the Toscana virus nucleoprotein**

Daniel Olal^1,3,^*, Alexej Dick^1^, Virgil L. Woods Jr^2^, Tong Liu^2^, Sheng Li^2^, Stephanie Devignot^3^, Friedemann Weber^3^, Erica Ollmann Saphire^4,5^ and Oliver Daumke^1,6,^*

^1^Max Delbrück Center for Molecular Medicine, Crystallography, Robert-Rössle-Strasse 10, 13125 Berlin, Germany

^2^Department of Medicine, University of California at San Diego, La Jolla, CA 92093

^3^Institute for Virology, Philipps-University Marburg, D-35043 Marburg, Germany

^4^Department of Immunology and Microbial Science, The Scripps Research Institute, La Jolla, CA USA

^5^The Skaggs Institute for Chemical Biology, The Scripps Research Institute, La Jolla, CA, USA

^6^Freie Universität Berlin, Biochemie, Takustrasse 6, 14195 Berlin, Germany

*To whom correspondence should be addressed. Tel: 0049-30-94063425; Fax: 0049-30-9406-3814; Email: oliver.daumke@mdc‑berlin.de

*Correspondence may also be addressed to Daniel Olal. Tel: 0049-30-94063275; F­ax: 0049-30-9406-3814; Email: daniel.olal@mdc-berlin.de

**Running title:** Structural transitions in the Toscana virus RNP

**Abstract**

Toscana virus is an emerging bunyavirus in Mediterranean Europe where it accounts for 80% of pediatric meningitis cases during the summer. The negative-strand RNA genome of the virus is wrapped around the virally encoded nucleoprotein N to form the ribonucleoprotein complex (RNP). We determined crystal structures of hexameric N alone (apo) and in complex with a nonameric single-stranded RNA. RNA is sequestered in a sequence-independent fashion in a deep groove inside the hexamer. At the junction between two adjacent copies of Ns, RNA binding induced an intersubunit rotation, which opened the RNA-binding tunnel and created a new assembly interface at the outside of the hexamer. Based on these findings, we suggest a structural model for how binding of RNA to N promotes the formation of helical RNPs, which are characteristic hallmark of many negative-strand RNA viruses.

**Introduction**

Toscana virus is an enveloped negative-strand RNA virus that belongs to the genus phlebovirus in the *Bunyaviridae* family. Members of this genus also include Rift Valley Fever virus (RVFV), Sandfly Fever Sicilian virus (SFSV), Severe Fever with Thrombocytopenia Syndrome (SFTS) virus and Uukuniemi virus. These viruses are etiological agents of a wide range of illnesses ranging from self-limiting febrile episodes to hemorrhagic fevers characterized by multi-organ failure and death . The sporadic outbreaks of these viruses, coupled with their widespread distribution across all continents, make them of great importance to public health. The genome of the virus is encoded in three segments of varying sizes known as the Large (L), Medium (M) and Small (S) segments that encode the viral polymerase, the envelope glycoprotein and the nucleoprotein (N), respectively . The S segment also encodes a virulence determinant known as non-structural small protein NSs in the sense direction.

The genome of phleboviruses, like other bunyaviruses, is tightly packed with N into ribonucleoprotein complexes (RNPs) . The RNP complex, rather than the free RNA, represents the transcription and replication template for the viral RNA-dependent RNA polymerase (RdRp). The RdRp docks on the RNP to synthesize viral mRNA and complementary antigenomic RNA .

Bunyaviruses replicate in the cytoplasm of host cells. For the initiation of transcription, RdRp employs an endonuclease activity to cleave and “snatch” the caps of host cell mRNA to prime viral transcription. This mechanism has been demonstrated for La Crosse virus and, by sequence comparison, inferred for all bunyaviruses .

Unlike other segmented viruses, members of the *Bunyaviridae* lack a matrix protein that connects the lipid envelope-associated glycoproteins to the RNP. Instead, in Uukuniemi virus, N interacts with the cytoplasmic tails of its cognate glycoproteins. This suggests that the bunyavirus N may, in part, fulfill the role of the missing matrix protein . The molecular architecture and RNA-binding mechanism of several negative-strand RNA viruses have recently been characterized. The N proteins of the *Rhabdoviridae* and *Paramyxoviridae* crystallized in rings, with the RNA bound in a tunnel formed by two lobes of the same monomer . The Lassa virus nucleoprotein has an exonuclease activity that is specific for dsRNA , further indicating that nucleoproteins can have a wider role beyond acting as scaffolds for the RdRp . Structures of orthobunyaviruses N, e.g. of Bunyamwera, Schmallenberg, Leanyer and La Crosse viruses , have revealed unique structural folds. In these cases, the RNA is bound in a positively charged cleft that is formed by two α-helical lobes. Oligomerization is mediated by both N- and C-terminal arms.

Recent studies have also provided insights into the mechanism of phlebovirus RNP assembly . The first structure of the RNA-free RVFV N showed a dimer, where an amino-terminal arm is folded back into the predicted RNA-binding pocket . This dimer is thought to represent an autoinhibited form of N. A second RNA-free structure of RVFV N showed a hexamer in the crystals . Crystal structures of RNA-bound multimers of RVFV N ranging from tetramers to hexamers showed how a poly-uridine RNA sequence is nonspecifically bound between two helical lobes .

In this study, we have elucidated structures of the hexameric RNA-free (apo) N and of hexameric N in complex with a single-stranded nonameric RNA. Our structural studies, combined with functional experiments, suggest a molecular mechanism of how RNA binding to N induces the transition from a hexameric ring to a helical RNP assembly.

**MATERIALS AND METHODS**

**Plasmids.** A codon-optimized cDNA of the Toscana virus N gene (Genbank accession code X53794) was commercially synthesized (Genscript) and supplied in the pUC57 vector. For bacterial expression as amino-terminal His_6_-fusion, the gene was cloned into pET15b or pET46-ek/LIC vectors (Novagen). RVFV N was expressed from the pET21 vector (Novagen). Plasmids for intracellular reconstitution of RVFV RNPs, pI.18-RVFV_L, pI.18-RVFV_N, pI.18-HA-PKRΔE7, and pHH21-RVFV-vMRen, were described previously . The pI.18-derived plasmids expressing RVFV N mutants Y30A, K74A, Y30A/K74A, Q198A, R185D, R185D/ D167A were constructed by site-specific mutagenesis. The firefly luciferase control plasmid pGL3 was purchased from Promega.

**Expression and Purification.** The recombinant plasmids were transformed into BL21 DE3 or BL21 DE3 Rosetta cells. Bacteria were cultured in LB medium at 37 °C until an OD_600_ of 0.4, followed by a temperature shift to 20 °C. The protein was produced for 18 h in the presence of 200 µM isopropyl β-D-1-thiogalactopyranoside (IPTG). Bacteria were collected by centrifugation and resuspended in lysis buffer containing 20 mM TRIS (pH 8), 10 mM imidazole, 300 mM NaCl and complete EDTA-free protease inhibitors, followed by cell disruption in a microfluidiser (Microfluidics). Lysates were cleared by centrifugation at 40,000 g for 30 min at 4 °C.

The supernatant was loaded on a 30 ml gravity flow column filled with 3 ml NiNTA resin pre-equilibrated with lysis buffer. The column was washed with 20 ml lysis buffer, followed by 25 ml washing steps with equilibration buffer (20 mM TRIS, pH 8.0, 500 mM NaCl) containing increasing concentrations of imidazole (10 mM, 25 mM, 75 mM). The protein was eluted from the column using 12.5 ml equilibration buffer containing 250 mM imidazole. N was further purified via size exclusion chromatography on a Sephadex 200 10/300 column (GE Healthcare) equilibrated with TRIS buffered saline (pH 7.5).

To remove heterogeneously bound RNA from *E. coli*, N bound to the NiNTA column was denatured by incorporating 8 M urea in the washing buffer. Bound RNA was washed off using high salt buffer (20 mM TRIS, pH 7.5, 500 mM NaCl), and N eluted by 20 mM TRIS, pH 7.5, 250 mM imidazole, 500 mM NaCl.

The fraction of bound RNA was determined by the A_260_/A_280_ ratio before and after refolding. The refolded N had an A_260_/A_280_ ratio of 0.6 while the native RNP complex purified directly from *E. coli* had a ratio of ~1.5 indicating about 10% bound nucleic acid.

The theoretical isoelectric point of N is 9.2 (ref. ). Cation exchange chromatography was used to further purify the refolded N and remove partially folded intermediates. To this end, the refolded protein was dialysed against 20 mM TRIS, pH 7.5, 50 mM NaCl and bound to a MonoS column (GE Healthcare). Protein was eluted using a salt gradient from 50 mM to 1000 mM NaCl.

**Circular Dichroism.** To verify the integrity of the refolded N, circular dichroism measurements were performed at room temperature using a protein concentration of 1 mg/ml in phosphate buffered saline, pH 7, in an Aviv 62A spectropolarimeter.

**Reconstitution of the RNP complex.** For reconstitution of a homogenous RNP, the refolded N was incubated at room temperature for 1 h with commercially synthesized nonameric RNA (UGUGUUUCU, IDT DNA Technologies Inc), at a molar ratio of 1:1. A final gel filtration step was added to remove unbound RNA. The stability of the reconstituted complex was monitored by gel filtration and also on a native gel. The A_260_/A_280_ ratio of the reconstituted RNP complex was 1.1, which corresponds to approximately 5% bound RNA.

**Electron Microscopy.** N or the indicated N mutants at a final concentration of 1.5 mg/ml were mixed with an *in vitro* transcribed 8 kb ssRNA comprising a GFP expression construct at a final concentration of 0.15 mg/ml. The sample was tenfold diluted with 20 mM TRIS, pH 7.5, 150 mM NaCl, and 15 µl of this solution was spotted onto carbon-coated grids. The sample was stained with 2% uranyl acetate and images were acquired on a Zeiss EM910 at magnifications between 12.5-31.5k.

**Crystallization and Data Collection.** Initial crystallization trials were carried out in 96-well formats using a Phoenix (Art Robbins Instruments) and Topaz system (Fluidigm Corp, South San Francisco, California, USA). Various commercially available screens, including PACT, PEG ion, Crystal (Qiagen) and Index Screen (Hampton Research) were used. Initial hits were translated to the hanging drop technique using a drop size of 1 µl + 1 µl at a protein concentration of 18-20 mg/ml. N crystals were grown in 6.5% PEG 3350, 0.1 M sodium malonate, pH 4.5-5.2, while RNP crystals were grown in 11% PEG 3350, 0.1 M sodium malonate, pH 4.5-5.2. Diffraction data of single crystals were collected at BESSY-II BL14.1 and processed using XDS and MOSFLM software .

The structure of Toscana virus N was solved by molecular replacement using a homology model of the RVFV N (PDB code 3OUO) calculated by the SWISS-MODEL server as a search model . Both Phaser and MOLREP programs were used . The phase problem for the RNP was solved by molecular replacement with the refined Toscana virus N as the search model using Phaser.

Both models were built with COOT alternating with refinement in PHENIX . For the RNP, positive density corresponding to the bound RNA was clearly visible after the first round of refinement. For both models, we employed dihedral restraints based on the high-resolution structure of N from RVFV. Strict NCS was used at the beginning of the refinement and then gradually released. TLS was used in the final rounds of refinement. Figures were prepared using PyMOL (The PyMOL Molecular Graphics System, Version 1.3, Schrödinger, LLC) and the pymol plugin VASco .

**Isothermal Titration Calorimetry (ITC).** ITC experiments were carried out at 8 °C in a VP-ITC or iTC200 (Microcal) using degassed 20 mM TRIS, pH 7.5, 150 mM NaCl. N was diluted and RNA resuspended in this buffer prior to the measurement. A 400 µM solution of single-stranded 6-9mer RNAs was titrated into a solution containing 30 µM apo N. Binding isotherms were fitted and equilibrium dissociation constants were calculated using the Microcal ORIGIN software.

**Deuterium exchange mass spectrometry.** To obtain optimal fragmentation sequence coverage of Toscana virus N, optimal quench conditions were established as previously described . Deuterium exchange experiments were carried out by mixing 2 µl of Toscana virus N stock solution with 6 µl D_2_O buffer (8.3 mM TRIS, pH 7.2, 150 mM NaCl in D_2_O) and incubation at 0 °C for 10 sec and 1000 sec. At the indicated time points, the exchange reaction was quenched by adding 12 µl of ice-cold optimal quench solution (0.05 M guanidinium hydrochloride, 15 mM TRIS (2-carboxyethyl)phosphine (TCEP), 16.6% glycerol, 0.8% formic acid (FA)), immediately frozen on dry ice and stored at –80 °C. The non-deuterated control samples and equilibrium-deuterated control samples were also prepared by mixing protein with H_2_O buffer (8.3 mM TRIS, pH 7.2, 150 mM NaCl in D_2_O) and equilibrium-deuterated buffer (0.8% FA in 99.9% D_2_O). The frozen samples were then thawed at 5 °C and passed over an immobilized pepsin column (16 µl bed volume) at a flow rate of 20 µl/min, and the resulting peptides were collected on a C18 trap for desalting and separated by a Magic C18AQ column (Michrom BioResources Inc.) using a linear gradient of acetonitrile from 6.4% to 38.4% over 30 min. Mass spectrometry (MS) analysis was performed using LCQ Classic mass spectrometer from Thermo Finnigan, with a capillary temperature of 200 °C. Data were acquired in both data-dependent MS/MS mode and MS1 profile mode, and the data analyzed by SEQUEST (Thermo Finnigan Inc.) and DXMS explorer (Sierra Analytics Inc., Modesto, CA).

**Minireplicon Assay.** Assays to measure activity of reconstituted RVFV RNPs were essentially performed as described . Briefly, subconfluent monolayers of 293 cells seeded in 12-well dishes were transfected with pI.18-RVFV_L, pI.18-RVFV_N (or mutants thereof), pI.18-HA-PKRΔE7, and pHH21-RVFV-vMRen (0.5 µg each), and the firefly luciferase control pGL3 (0.05 µg), using Nanofectin (PAA). At 24 h post-transfection, cell extracts were tested for Renilla and firefly luciferase activities using the Dual luciferase method (Promega).

**RESULTS**

**Structure and oligomerization of Toscana virus N**

N of Toscana virus was expressed in *E. coli* and purified via affinity chromatography. The resulting protein was associated with *E. coli* nucleic acids, as apparent by the observed A_260_/A_280_ ratio of 1.5. This preparation yielded protein crystals that showed only weak, anisotropic diffraction.

Using an unfolding/refolding protocol, nucleic acid‑free N was obtained, as judged from the A_260/_A_280_ ratio of 0.6 (Supplementary Figure S1A). Correct folding of the protein was confirmed by circular dichroism (CD) spectroscopy (Supplementary Figure S1B). Analytical gel filtration experiments combined with static light scattering indicated a tendency of the protein to self-assemble even in the absence of RNA (Figure 1A), unlike the N of the closely related RVFV, which exists as a dimer in solution .

It was previously reported that Ns of bunyaviruses bind with high affinity to the highly conserved 5’ and 3’ sequences of their genomic RNA segments . In agreement with this, N of Toscana virus bound to a nonameric single stranded RNA sequence corresponding to the 3’ genomic RNA consensus with an affinity of 560 nM in isothermal titration calorimetry (ITC) experiments (Supplementary Figure S2A, Table 1). Only a weak binding signal was obtained for a 9mer RNA with the 5' genomic RNA consensus sequence (Supplementary Figure S2B). Interestingly, Toscana virus N bound with highest affinity (K_d_=260 nM) to a control 9mer RNA corresponding to the reverted 3' consensus sequence (termed oligo1, Figure 1B). In these titrations, a binding stoichiometry of 0.5 was derived, indicating that only every second N bound an RNA molecule. The observed RNA binding affinities were approximately 10-fold lower compared to those reported for RVFV Ns . This difference is likely related to the lower salt concentration and the longer RNA oligomers used in the previous study since these authors reported similar RNA binding affinities for Toscana and RVFV N in their experimental setup. Furthermore, RVFV N bound to oligo1 with an almost identical affinity (K_D_= 420 nM) compared to Toscana virus N in ITC experiments (Supplementary Figure S2H).

To further analyze the sequence-specificity of N, base pair substitutions were introduced in oligo1. The exchange of guanine 2 (G2) to adenine did not have a significant effect on the binding affinity (Supplementary Figure S2C). Furthermore, the affinity for a 9mer polyU sequence was only slightly reduced compared to the original 9mer sequence (Supplementary Figure S2D), indicating a certain degree of plasticity in RNA recognition.

We then reconstituted Toscana virus RNPs *in vitro* using an 8000 bp single stranded RNA. By negative-stain electron microscopy, we observed filamentous RNP scaffolds with a diameter of 10 - 12.5 nm that may have an underlying helical sub-pattern (Figure 1C). The morphology of these RNPs was reminiscent of that of orthobunyavirus RNPs , but differed from those reported for RVFV that showed a more extended conformation .

Crystals of N in the absence and presence of the control 9mer RNA were obtained and diffracted to a maximal resolution of 3.3 Å and 2.6 Å, respectively (Table 2). The phase problem was solved by molecular replacement. The apo structure contained 12 molecules of N in the asymmetric unit, which were organized in two hexameric rings. The RNA-containing N crystals had 24 N and 12 RNA molecules in the asymmetric unit, which were arranged in four almost identical hexameric rings (Supplementary Figures S3A and B). The use of non-crystallographic symmetry improved electron density during refinement and allowed the unambiguous modeling of the complete 9mer RNA in the RNP crystals. The apo structure was refined to an R_work_/R_free_ of 24.7% / 27.5% and the RNP structure to an R_work_/R_free_ of 22.5% / 25.8% (Table 2). As in the RVFV N structure , each N monomer consisted of a core domain comprised of two α-helical lobes (the upper and lower lobe), and an amino-terminal α-helical arm which was divided by a short linker, hinge II, in α1 and α2 (Figure 1D and Supplementary Figure S4).

In the hexameric rings of the apo structure, an almost perfect six-fold symmetry between the six monomers was observed (Figure 1E). Oligomerization was driven by the amino-terminal arm, which bound into a hydrophobic groove of the neighboring monomer at the outside of the hexamer (pocket I and II in Figures 1E and F, Supplementary Figure S3). This interaction constituted the major oligomerization contact, as evident by the buried surface area of 1530 Å^2^ (the total buried surface between two monomers is 1690 Å^2^). The interaction of helix α1 with the neighboring monomer included hydrogen bonds and hydrophobic contacts, and a salt bridge between Lys83 and Glu29 of the opposing monomer (Supplementary Figure S5A). A similar oligomerization mode has been reported for RVFV RNPs . It also agrees with a recent structure of hexameric RNA-free Toscana virus N which was published during the preparation of this manuscript .

To probe dynamic features of the hexamer, hydrogen/deuterium exchange (HDX) experiments were performed. These studies showed that, in particular residues 16-24, corresponding to hinge II of the amino-terminal arm, underwent rapid hydrogen exchange in the N hexamer (Supplementary Figure S5B), pointing to a highly dynamic association of this region in solution.

**Sequence-independent RNA binding in Toscana virus N**

Also in the RNA-bound RNP crystals, oligomerization of N into hexamers was mediated by the amino-terminal α-helical arm, which filled the hydrophobic groove of the core domain at the outside of hexamer (Figure 2A). In agreement with our ITC data, only every second N bound an RNA molecule, whereas the central RNA-binding cleft of the neighboring monomer was empty. In all 12 RNA-bound Ns of the asymmetric unit, the 9mer RNA occupied a defined position in a positively charged cleft at the inside of the hexameric ring, where the first and last residue of the RNA could clearly be distinguished in the electron density (Figure 2A, 2B and Supplementary Figure S6). This argues for a preferred binding mode of the 9mer RNA to the N hexamer.

The orientation of the RNA is evident from the asymmetric distribution of the two guanine (G) bases at position 2 and 4 which could be discriminated from the remaining pyrimidine bases in the 9mer RNA (Figure 2B). The RNA bases are deeply buried in the interior of the RNA binding groove and not accessible from the outside. Some of the bases stacked against each other, for example G4 and U5, and C7, U8 and U9 (Figures 2C and 2D). All bases projected towards the protein, with the exception of G4 which pointed towards the interior of the hexameric ring. Tyr32 stacks against the base of G2, while Tyr32 of the neighboring subunit stacks against the base of uridine 9 (U9) (Figure 2C). In this way, each second N sequesters 7 ribonucleotides, while 2 overlapping nucleotides are bound by neighboring subunits. The resulting steric restraints may explain why only every second N is occupied by RNA. In addition to Tyr32, other residues contacted the RNA bases. For example, Phe35 stacks against U3, and Ser211, Ser110 and Arg191 interacted via side chain interactions with the bases of U1, U6 and C7, respectively. Thr201 interacts with the base of G4 via a main-chain interaction (Figure 2C).

The phosphate backbone of the 9mer RNA is recognized by several positively charged and polar residues. For example, Arg111 and Lys72 formed salt bridges to the phosphates of G4 and U6, respectively. Interestingly, Lys204 and Lys79 formed hydrogen bonds to the phosphate backbone at the 5' end of the RNA whereas they do not interact with the phosphate backbone at the 3' RNA end (Figure 2C).

Assuming an identical conformation of the phosphate backbone, we modeled the nonameric sequence of the 5' non-coding region (oligo3 in Table 1) in the RNA-binding groove (Supplementary Figure S7). Due to the higher abundance of purine bases in oligo3, steric clashes were predicted. This may explain the reduced affinity of this sequence to N.

During the preparation of this manuscript, tetra-, penta- and hexameric structures of the RVFV N bound to 28 - 36mer polyU RNA sequence were reported . A comparison with our RNP structure indicates that the gross features of RNA recognition are conserved between Toscana virus and RVFV. Tyr32 has a direct counterpart in RVFV N (Tyr30), which similarly stacks with the guanine and uridine base, respectively. Also the phosphate backbone interacts in a similar fashion with the protein in both structures; for example, Lys72 and Arg111 in Toscana virus N are conserved in both viruses and form similar contacts to the phosphates. However, species-dependent differences exist. In RVFV N, Arg70 contacts the phosphate backbone whereas the corresponding alanine in Toscana virus N is not involved in RNA binding. Vice versa, Lys204 at the 5' RNA end in our RNP forms a salt bridge to the phosphate of RNA residue 1 whereas the corresponding glutamine residue in the RVFV RNP structure does not participate in RNA binding (see also below). Arg99 in the RVFV N complex makes a prominent contact with Tyr32 and additionally contacts the phosphate backbone. Somewhat surprisingly, the corresponding residue in Toscana virus N, Arg104, is not involved in RNA binding in our model. Interestingly, G4 in the Toscana virus N structure is rotated compared to the corresponding uridine in RVFV N and faces the solvent channel, which might represent a mechanism to allow base plasticity at this position. Our results suggest that the general mode of RNP assembly is conserved in phleboviruses, while minor species-specific differences may exist.

**The RNA binding tunnel**

The availability of both apo and RNA-bound N structures allow for the characterization of conformational changes associated with RNA-binding. In the apo- and the RNA-bound forms, the core domains of N excluding the amino-terminal arm superimpose with a root mean square deviation (rmsd) of 0.4 Å indicating that RNA binding induces only minor conformational changes within the α-helical core. However, the amino-terminal arm of the RNA-bound subunits is considerably displaced against the N core domain when compared to the apo structure or the RNA-free Ns in the RNP (Figure 3A and Supplementary Figure S6). In the apo N structure, Tyr32 from hinge I, Lys204 from the lower lobe and Lys79 from the upper lobe of the adjacent subunit are in close proximity to each other and cap the RNA binding tunnel (Figure 3B). Due to their direct RNA interaction (Figure 3A), Tyr32 and Lys79 become separated from Lys204 by approximately 15 Å (Figure 3C). Interestingly, this extensive separation is only observed at the intersubunit junction at the 5', but not at the 3' RNA end, where Lys79 and Lys204 do not reach the phosphate backbone (Figure 2C and Supplementary Figure S8).

Mutagenesis was performed in order to assess the importance of Tyr32, Lys79 and Lys204 for RNA binding. The Y32A mutant bound the 9mer RNA with 6-fold lower affinity (K_d_ = 1.6 µM) compared to wild-type N (Figure 3D). In contrast, the K79A and K204A mutation did not significantly affect the RNA binding affinity (Figure 3D). Interestingly, the simultaneous mutation of both Tyr32 and Lys79 significantly diminished the RNA binding affinity (K_d_ = 6 µM), more than the single Y32A mutation, suggesting that these two adjacent residues cooperate in RNA binding. All three single mutants were able to form RNPs *in vitro* using a synthetic ssRNA as a template, although RNPs formed by the K204A mutant appeared somewhat shorter compared to wild-type (Figure 3E). The Y32A/K79A double mutant assembled into structures more reminiscent of aggregates than functional filaments, suggesting some problems in proper RNP assembly.

The importance of these residues for viral RNP activity was assessed using a system for intracellular reconstitution of viral RNPs from transfected cDNA plasmids, a so-called minireplicon assay. Since this assay has not been established for Toscana virus, we initially tested a heterologous approach using the RVFV RdRp and N of Toscana virus to drive the replication of a reporter gene . However, we were unable to detect any reporter activity in this hybrid system (data not shown).

We then switched to the well-established RVFV minireplicon system and introduced mutations to equivalent residues within the RVFV N . While Tyr32 and Lys79 are highly conserved in phleboviruses (corresponding to Tyr30 and Lys74 in RVFV, Supplementary Figure S4), Lys204 in Toscana virus N is replaced by Gln198 in RVFV, which similarly could contact the phosphate backbone. Surprisingly, mutating the invariant Tyr30 or Lys74 to alanine did not significantly hamper reporter activity (Figure 3F). However, simultaneous mutation of both conserved residues resulted in a stable N, which was nonetheless inactive. This suggests that Tyr30 and Lys74 work in concert to mediate viral replication. The Q198A mutant exhibited a reduced activity relative to wild-type, possibly pointing to an important function in viral replication (Figure 3F). However, Western blot analyses showed that Q198A was largely undetectable, indicating a general instability.

**Coupling of RNA binding to an inter-subunit rearrangement**

The four hexameric rings in the asymmetric unit of the RNA-bound N hexamers were stacked in a bottom-to-bottom assembly, e.g. in the opposite orientation as in the apo structure (Supplementary Figure S3). Every second N monomer was laterally displaced relative to the neighboring N leading to a staggered assembly of the hexameric ring. In this way, the six-fold symmetry in the N hexamer was broken, resulting in three-fold symmetry of the RNP (Figures 4A and 4B). Considerable differences were apparent in the inter-subunit assembly of N at the 5’ and 3’ ends of the 9mer RNA molecules. In the following, we refer to the N interplay at the 5’ RNA end, which appears to represent a functional assembly akin to helical RNP structures (see below).

Analysis of the inter-subunit assembly revealed that the position of Tyr32, and in fact the whole N-terminal arm, relative to the associating N was almost identical in the apo- and RNA-bound forms (Figure 4C). However, the simultaneous binding of Lys204 and Lys79 of the adjacent subunit to the phosphate backbone of the RNA led to a 45° rotation between the subunits, with Tyr32 working as a pivot point (Figure 4C and 4D). This domain movement created a new inter-subunit interface of 480 Å^2^ at the outside of the hexamer, which did not involve the amino-terminal arm (Figure 4E and 4F). It included several hydrogen bonds and a salt bridge between Asp173 and Arg191 from two opposing subunits, which moved 17 Å towards each other. At the inter-subunit junction at the 3’ RNA end, Lys204 and Lys79 did not bind RNA and occupied a similar position as in the apo form relative to the neighboring monomer (Supplementary Figure S8). Also in the RNA- and DNA-bound ring-like RNP structures of RVFV, Gln198 does not bind to nucleic acids. Consequently, no marked inter-subunit rotation is observed in these structures and no additional interface is formed (Supplementary Figure S9).

To clarify the importance of this new interface, we introduced mutations and analyzed the consequence for viral transcription and RNP production (Figure 4G). The single point mutant R185D was expressed similarly to wild-type, but exhibited greatly reduced activity in the reporter gene assay. The double mutant, D167A/R185D was unstable and, accordingly, also did not show reporter activity. These data are consistent with an important role of this interface for RNP activity.

To further characterize this domain rearrangement, binding of shorter RNA oligomers to N was evaluated. As expected, consecutive shortening of the RNA gradually reduced the affinity for N (Supplementary Figures 2E, 2F and 2G). Surprisingly, the binding stoichiometries of the shorter RNA oligomers (6 - 8mers) did not significantly differ from the 9mer RNAs (n= 0.4 - 0.5). Since each N binds 7 RNA bases, the 6mer RNA would have enough space to bind to adjacent Ns in the hexameric ring. Our data suggest that RNA binding and the associated structural rearrangements of adjacent subunits prevents binding of a second RNA molecule in the context of a hexameric ring.

**Model for the helical RNPs**

Our EM analysis suggested that Toscana virus N forms 10 nm RNP fibers upon interaction with longer RNAs (Fig. 1C). To obtain structural insights into RNP formation, we applied the observed intersubunit shift observed at the 5'-RNA end to all consecutive subunits. This transformation resulted in a left-handed N helix with an outer diameter of 10 nm (Figure 6). Each turn of this helix contained 5-6 N corresponding to the sequestration of 35-42 RNA nucleotides. In this model, long RNA sequences could be encapsidated in a continuous RNA binding tunnel at the inside of the RNP involving consecutive Ns.

**DISCUSSION**

Phleboviruses are important human pathogens, and studying their mechanism of RNA encapsidation and replication is of vital importance. In the current study, we analyzed structures of uncomplexed and RNA-bound N of Toscana virus. These structures show that the gross features of oligomerization and RNA binding in Ns are conserved amongst phleboviruses . Oligomerisation is mediated by an amino-terminal arm that binds in a hydrophobic pocket of the next monomer. RNA, in turn, is sequestered in a positively charged cavity at the inside of the oligomer, in a mostly sequence-independent fashion. However, it has been unclear how the hexameric planar architectures of the apo Ns of RVFV and Toscana virus are compatible with binding of longer RNA sequences. Also the RNA- and DNA-bound structures of RVFV RNPs show ring-like architectures, which do not explain how longer nucleotide sequences can be bound .

Here, we describe a molecular mechanism how RNA binding can be coupled to an intersubunit movement in the hexameric N inducing the transition from a ring-like to a helical RNP. Central to this intersubunit movement is the interplay between Tyr32 and Lys204 in one subunit, Lys79 in the adjacent subunit and their interactions with RNA. In the absence of RNA, Tyr32, Lys204 and Lys79 localize closely together in the RNA-binding tunnel. At the 5' end of our 9mer RNA, they become separated upon RNA binding. Tyr32 and Lys79 cooperate to bind the RNA from the upper side, whereas Lys204 contacts the phosphate backbone from the lower lobe. In this way, the RNA-binding cleft is opened and a continuous RNA binding tunnel is created. This situation is somewhat reminiscent of Lassa virus RNPs where a flexible loop regulates the open and closed conformations of the RNA tunnel . The conformational changes resulting from RNA binding are then translated into an inter-subunit rotation creating a new assembly interface at the outside of the oligomer. Mutations in this new interface reduce the transcriptional activity, as apparent in the minireplicon assay. We propose that the observed intersubunit architecture at our 5' 9mer RNA represents the architecture of every intersubunit junction in a functional RNP. The almost identical assembly of all twelve intersubunit junctions in four independent RNPs in the asymmetric unit argues for a defined state of the intersubunit junction, rather than a random displacement of Ns in the crystal lattice. Most importantly, such architecture would allow binding of longer RNA sequences in a continuous helical RNP oligomer. To our knowledge, this is the first example describing the molecular mechanism of how RNA binding induces transition from a planar ring to a helical structure in an RNP assembly.

Why is such transition not observed in the crystal structures of RVFV RNPs or at the 3' end of the RNA molecule? Also in the RVFV RNPs, the three residues in the intersubunit junction (Tyr30, Lys74 and Gln198) separate upon RNA binding. However, Gln198 does not contact the RNA and, consequently, no inter-subunit rotation is observed. The RNA and DNA sequences used for the crystallization of the RVFV RNP symmetrically fit into one ring at the inside of the RNP. We speculate that, in these cases, a closed ring structure might be more stable than an open helix turn and therefore be favored for crystallization. Similarly, Lys204 and Lys79 do not contact the RNA backbone at the 3' RNA end of our Toscana virus RNP and, consequently, no major rotation is observed. The 9mer RNA may be too short at this side and/or the induction of a similar shift as at the 5' RNA end would induce an open ring and therefore be energetically unfavored. Furthermore, it can be envisaged that the proposed intersubunit shift is energetically not favoured over the planar architecture, since we do not observe it in the apo N hexamer. Instead, it may be driven by RNA binding. This may also explain why the K204A and K79A mutants still bind with similar affinity compared to wild-type N to our 9mer RNA - the loss of binding energy due to these mutations may be compensated by a reduced intersubunit shift. In support of such hypothesis, the *in vitro* reconstituted RNPs of the K204A mutant appear shorter compared to wild-type, and we see reduced activity of the corresponding RVFV N proteins K74A/Y30A in the minireplicon assays.

It has been known for some time that phlebo- and tenuiviruses are evolutionarily related . Both have multipartite genomes with identical 5’ termini nucleotides (5’-ACACAAAG) and complementary 3’ nucleotides. Their segmented genomes are both negative and ambisense and the encoded proteins show significant sequence and structural similarity. Accordingly, the phlebovirus and tenuivirus N belong to the same protein family (Pfam 05733). Furthermore, structural predictions reveal significant homology between the Ns of Toscana and Rice hoja blanca and maize stripe tenuiviruses (Supplementary Figure S10A and 10B) . Interestingly, tenuivirus RNPs can assemble into helical and superhelical structures (Supplementary Figure S10C) . Also the reported diameter of the tenuiviruses (8 nm) agrees well with the diameter of our RNP model helix (10 nm). We therefore envisage that our proposed mechanism for the helical assembly induced by RNA sequestration in Toscana virus RNPs is applicable to a wider range of viruses.

**ACCESSION NUMBERS**

Coordinates and structure factors for the Toscana virus N and N-RNA complex have been deposited in the Protein Data Bank under accession codes 4csg and 4csf.

**SUPPLEMENTARY DATA**

Supplementary Data are available at NAR online: Supplementary Figures 1-10

**ACKNOWLEDGEMENTS**

We are grateful to Drs. Uwe Müller and Manfred Weiss and the staff of BESSY for their excellent assistance in data collection and Bettina Purfürst for her help with the EM. We also would like to thank Drs. Katja Fälber, Yvette Roske and Georg Kochs for helpful discussions, Dr. Stephen Marino for *in vitro* transcribed ssGFP RNA and Julia Wulle for expert technical assistance.

**FUNDING**

Deutsche Forschungsgemeinschaft (DFG) [DFG; DA 1127/1 to O.D.], [DFG; SFB 1021 / B06 to F.W.]). This work is part of the CCH Fever Network (Collaborative Project) supported by the European Commission under the Health Cooperation Work Programme of the 7th Framework Programme (grant agreement no. 260427 to F.W.). An Investigators in the Pathogenesis of Infectious Disease award from the Burroughs Wellcome Fund to E.O.S.

REFERENCES

1.	Valassina, M., Cusi, M.G. and Valensin, P.E. (1996) Rapid identification of Toscana virus by nested PCR during an outbreak in the Siena area of Italy. *J Clin Microbiol*, **34**, 2500-2502.

2.	Valassina, M., Cusi, M.G. and Valensin, P.E. (2003) A Mediterranean arbovirus: the Toscana virus. *J Neurovirol*, **9**, 577-583.

3.	Le May, N., Dubaele, S., Proietti De Santis, L., Billecocq, A., Bouloy, M. and Egly, J.M. (2004) TFIIH transcription factor, a target for the Rift Valley hemorrhagic fever virus. *Cell*, **116**, 541-550.

4.	Yu, X.J., Liang, M.F., Zhang, S.Y., Liu, Y., Li, J.D., Sun, Y.L., Zhang, L., Zhang, Q.F., Popov, V.L., Li, C. *et al.* (2011) Fever with thrombocytopenia associated with a novel bunyavirus in China. *N Engl J Med*, **364**, 1523-1532.

5.	Le May, N., Gauliard, N., Billecocq, A. and Bouloy, M. (2005) The N terminus of Rift Valley fever virus nucleoprotein is essential for dimerization. *J Virol*, **79**, 11974-11980.

6.	Ruigrok, R.W. and Crepin, T. (2010) Nucleoproteins of negative strand RNA viruses; RNA binding, oligomerisation and binding to polymerase co-factor. *Viruses*, **2**, 27-32.

7.	Reguera, J., Weber, F. and Cusack, S. (2010) Bunyaviridae RNA polymerases (L-protein) have an N-terminal, influenza-like endonuclease domain, essential for viral cap-dependent transcription. *PLoS Pathog*, **6**, e1001101.

8.	Morin, B., Coutard, B., Lelke, M., Ferron, F., Kerber, R., Jamal, S., Frangeul, A., Baronti, C., Charrel, R., de Lamballerie, X. *et al.* The N-terminal domain of the arenavirus L protein is an RNA endonuclease essential in mRNA transcription. *PLoS Pathog*, **6**, e1001038.

9.	Overby, A.K., Pettersson, R.F. and Neve, E.P. (2007) The glycoprotein cytoplasmic tail of Uukuniemi virus (Bunyaviridae) interacts with ribonucleoproteins and is critical for genome packaging. *J Virol*, **81**, 3198-3205.

10.	Overby, A.K., Pettersson, R.F., Grunewald, K. and Huiskonen, J.T. (2008) Insights into bunyavirus architecture from electron cryotomography of Uukuniemi virus. *Proc Natl Acad Sci U S A*, **105**, 2375-2379.

11.	Tawar, R.G., Duquerroy, S., Vonrhein, C., Varela, P.F., Damier-Piolle, L., Castagne, N., MacLellan, K., Bedouelle, H., Bricogne, G., Bhella, D. *et al.* (2009) Crystal structure of a nucleocapsid-like nucleoprotein-RNA complex of respiratory syncytial virus. *Science*, **326**, 1279-1283.

12.	Albertini, A.A., Wernimont, A.K., Muziol, T., Ravelli, R.B., Clapier, C.R., Schoehn, G., Weissenhorn, W. and Ruigrok, R.W. (2006) Crystal structure of the rabies virus nucleoprotein-RNA complex. *Science*, **313**, 360-363.

13.	Hastie, K.M., Liu, T., Li, S., King, L.B., Ngo, N., Zandonatti, M.A., Woods, V.L., Jr., de la Torre, J.C. and Saphire, E.O. (2011) Crystal structure of the Lassa virus nucleoprotein-RNA complex reveals a gating mechanism for RNA binding. *Proc Natl Acad Sci U S A*, **108**, 19365-19370.

14.	Hastie, K.M., Kimberlin, C.R., Zandonatti, M.A., MacRae, I.J. and Saphire, E.O. (2011) Structure of the Lassa virus nucleoprotein reveals a dsRNA-specific 3' to 5' exonuclease activity essential for immune suppression. *Proc Natl Acad Sci U S A*, **108**, 2396-2401.

15.	Qi, X., Lan, S., Wang, W., Schelde, L.M., Dong, H., Wallat, G.D., Ly, H., Liang, Y. and Dong, C. (2010) Cap binding and immune evasion revealed by Lassa nucleoprotein structure. *Nature*, **468**, 779-783.

16.	Hastie, K.M., King, L.B., Zandonatti, M.A. and Saphire, E.O. (2012) Structural basis for the dsRNA specificity of the Lassa virus NP exonuclease. *PLoS One*, **7**, e44211.

17.	Li, B., Wang, Q., Pan, X., Fernandez de Castro, I., Sun, Y., Guo, Y., Tao, X., Risco, C., Sui, S.F. and Lou, Z. (2013) Bunyamwera virus possesses a distinct nucleocapsid protein to facilitate genome encapsidation. *Proc Natl Acad Sci U S A*, **110**, 9048-9053.

18.	Ariza, A., Tanner, S.J., Walter, C.T., Dent, K.C., Shepherd, D.A., Wu, W., Matthews, S.V., Hiscox, J.A., Green, T.J., Luo, M. *et al.* (2013) Nucleocapsid protein structures from orthobunyaviruses reveal insight into ribonucleoprotein architecture and RNA polymerization. *Nucleic Acids Res*, **41**, 5912-5926.

19.	Dong, H., Li, P., Bottcher, B., Elliott, R.M. and Dong, C. (2013) Crystal structure of Schmallenberg orthobunyavirus nucleoprotein-RNA complex reveals a novel RNA sequestration mechanism. *Rna*, **19**, 1129-1136.

20.	Niu, F., Shaw, N., Wang, Y.E., Jiao, L., Ding, W., Li, X., Zhu, P., Upur, H., Ouyang, S., Cheng, G. *et al.* (2013) Structure of the Leanyer orthobunyavirus nucleoprotein-RNA complex reveals unique architecture for RNA encapsidation. *Proc Natl Acad Sci U S A*, **110**, 9054-9059.

21.	Reguera, J., Malet, H., Weber, F. and Cusack, S. (2013) Structural basis for encapsidation of genomic RNA by La Crosse Orthobunyavirus nucleoprotein. *Proc Natl Acad Sci U S A*, **110**, 7246-7251.

22.	Ferron, F., Li, Z., Danek, E.I., Luo, D., Wong, Y., Coutard, B., Lantez, V., Charrel, R., Canard, B., Walz, T. *et al.* (2011) The hexamer structure of Rift Valley fever virus nucleoprotein suggests a mechanism for its assembly into ribonucleoprotein complexes. *PLoS Pathog*, **7**, e1002030.

23.	Raymond, D.D., Piper, M.E., Gerrard, S.R., Skiniotis, G. and Smith, J.L. (2012) Phleboviruses encapsidate their genomes by sequestering RNA bases. *Proc Natl Acad Sci U S A*, **109**, 19208-19213.

24.	Raymond, D.D., Piper, M.E., Gerrard, S.R. and Smith, J.L. (2010) Structure of the Rift Valley fever virus nucleocapsid protein reveals another architecture for RNA encapsidation. *Proc Natl Acad Sci U S A*, **107**, 11769-11774.

25.	Habjan, M., Penski, N., Wagner, V., Spiegel, M., Overby, A.K., Kochs, G., Huiskonen, J.T. and Weber, F. (2009) Efficient production of Rift Valley fever virus-like particles: The antiviral protein MxA can inhibit primary transcription of bunyaviruses. *Virology*, **385**, 400-408.

26.	Gasteiger, E., Gattiker, A., Hoogland, C., Ivanyi, I., Appel, R.D. and Bairoch, A. (2003) ExPASy: The proteomics server for in-depth protein knowledge and analysis. *Nucleic Acids Res*, **31**, 3784-3788.

27.	Lundstrom, K. (2003) Semliki Forest virus vectors for large-scale production of recombinant proteins. *Methods in molecular medicine*, **76**, 525-543.

28.	Kabsch, W. (2010) Xds. *Acta Crystallogr D Biol Crystallogr*, **66**, 125-132.

29.	Winn, M.D., Ballard, C.C., Cowtan, K.D., Dodson, E.J., Emsley, P., Evans, P.R., Keegan, R.M., Krissinel, E.B., Leslie, A.G., McCoy, A. *et al.* (2011) Overview of the CCP4 suite and current developments. *Acta Crystallogr D Biol Crystallogr*, **67**, 235-242.

30.	Kiefer, F., Arnold, K., Kunzli, M., Bordoli, L. and Schwede, T. (2009) The SWISS-MODEL Repository and associated resources. *Nucleic Acids Res*, **37**, D387-392.

31.	Vagin, A. and Teplyakov, A. (2010) Molecular replacement with MOLREP. *Acta Crystallogr D Biol Crystallogr*, **66**, 22-25.

32.	McCoy, A.J., Grosse-Kunstleve, R.W., Adams, P.D., Winn, M.D., Storoni, L.C. and Read, R.J. (2007) Phaser crystallographic software. *J Appl Crystallogr*, **40**, 658-674.

33.	Emsley, P. and Cowtan, K. (2004) Coot: model-building tools for molecular graphics. *Acta Crystallogr D Biol Crystallogr*, **60**, 2126-2132.

34.	Adams, P.D., Afonine, P.V., Bunkoczi, G., Chen, V.B., Davis, I.W., Echols, N., Headd, J.J., Hung, L.W., Kapral, G.J., Grosse-Kunstleve, R.W. *et al.* (2010) PHENIX: a comprehensive Python-based system for macromolecular structure solution. *Acta Crystallogr D Biol Crystallogr*, **66**, 213-221.

35.	Steinkellner, G., Rader, R., Thallinger, G.G., Kratky, C. and Gruber, K. (2009) VASCo: computation and visualization of annotated protein surface contacts. *BMC Bioinformatics*, **10**, 32.

36.	Englander, J.J., Del Mar, C., Li, W., Englander, S.W., Kim, J.S., Stranz, D.D., Hamuro, Y. and Woods, V.L., Jr. (2003) Protein structure change studied by hydrogen-deuterium exchange, functional labeling, and mass spectrometry. *Proc Natl Acad Sci U S A*, **100**, 7057-7062.

37.	Klemm, C., Reguera, J., Cusack, S., Zielecki, F., Kochs, G. and Weber, F. (2013) Systems to establish bunyavirus genome replication in the absence of transcription. *J Virol*, **87**, 8205-8212.

38.	Schmaljohn, C.S. and Patterson, J.L. (1990) *Bunyaviridae and their replication. Part	II: Replication of Bunyaviridae, in Fields Virology* 2nd ed. Raven Press, New York.

39.	Zheng, W. and Tao, Y.J. (2013) Genome encapsidation by orthobunyavirus nucleoproteins. *Proc Natl Acad Sci U S A*, **110**, 8769-8770.

40.	Accardi, L., Prehaud, C., Di Bonito, P., Mochi, S., Bouloy, M. and Giorgi, C. (2001) Activity of Toscana and Rift Valley fever virus transcription complexes on heterologous templates. *J Gen Virol*, **82**, 781-785.

41.	Estabrook, E.M., Tsai, J. and Falk, B.W. (1998) In vivo transfer of barley stripe mosaic hordeivirus ribonucleotides to the 5' terminus of maize stripe tenuivirus RNAs. *Proc Natl Acad Sci U S A*, **95**, 8304-8309.

42.	Jaroszewski, L., Rychlewski, L., Li, Z., Li, W. and Godzik, A. (2005) FFAS03: a server for profile--profile sequence alignments. *Nucleic Acids Res*, **33**, W284-288.

43.	Kelley, L.A. and Sternberg, M.J. (2009) Protein structure prediction on the Web: a case study using the Phyre server. *Nature protocols*, **4**, 363-371.

44.	Espinoza, A.M., Hernandez, M., Pereira, R., Falk, B. and Medina, V. (1992) In situ immunogold labeling analysis of the rice hoja blanca virus nucleoprotein and major noncapsid protein. *Virology*, **191**, 619-627.

45.	Davis, I.W., Leaver-Fay, A., Chen, V.B., Block, J.N., Kapral, G.J., Wang, X., Murray, L.W., Arendall, W.B., 3rd, Snoeyink, J., Richardson, J.S. *et al.* (2007) MolProbity: all-atom contacts and structure validation for proteins and nucleic acids. *Nucleic Acids Res*, **35**, W375-383.

**Figures**

**Figure 1: Structure and oligomerization of the Toscana virus N protein**

(A) Gel filtration coupled to right angle light scattering (RALS) analysis of the Toscana virus N. UV absorbance is shown in red and refers to the left y-axis, while the apparent molecular mass (in multiples of the molecular weight of the monomer) is shown in green and refers to the right y-axis.

(B) ITC experiments were carried out in 20 mM TRIS, pH 7.5, 150 mM NaCl at 8 °C to determine equilibrium dissociation constants of the nonameric single-stranded RNA oligo1 (UGUGUUUCU, Table 1) to the hexameric N (K_d_ = 260 nM ± 20 nM, n= 0.49 ± 0.01). Errors represent standard deviations derived from the fittings.

(C) Electron micrographs of negatively stained, 8000 bp single-stranded RNA, Toscana virus N and *in vitro* reconstituted Toscana virus RNP particles, respectively.

(D) Structure of the Toscana virus N, which is comprised of a two-lobed core domain and an amino-terminal arm comprising α1 and α2. These helices are linked to the core domain by hinge I.

(E) Ribbon representation of the hexameric Toscana virus N showing that the N-terminal arm mediates oligomerization by wrapping around the neighboring subunit. The six-fold symmetry axis of the N hexamer is indicated.

(F) Hydrophobic surface representation in which hydrophobic residues are shown in green and polar residues in yellow. The amino-terminal arm of the neighboring subunit is shown in stick representation. Ile23, Trp26 and Phe30 are inserted in pocket I while Leu11, Phe13 and Leu14 bind into pocket II.

**Figure 2: RNA binding to the N hexamer**

(A) Surface representation of the Toscana virus RNP complex showing three RNA molecules in red bound to the inside of the hexameric ring. Binding of RNA induces a change from a six-fold to a three-fold symmetry (triangle).

(B) 2F_o_-F_c_ map contoured at 2σ showing electron density for the bound RNA oligomer. The asymmetric sequence allowed unambiguous fitting of the nonameric RNA into the electron density.

(C) Cartoon representation showing selected contacts of N with the RNA. The RNA is capped on either side by Tyr32 of adjacent monomers. Residues depicted in white belong to the adjacent subunits (see Fig. 2A for chain numbering).

(D) Schematic diagram of the orientation of the RNA bases in the RNA binding groove. RNA bases are recognized by both main and side-chain contacts.

**Figure 3: Conformational changes in the RNA binding tunnel**

(A) Superposition of an apo (brown) and RNA-bound (blue) N monomer (bound RNA is not shown). In these two structures, the amino-terminal arm is displaced relative to the core domain.

(B) Cartoon representation showing the Tyr32-Lys79-Lys204 triad, which is situated in the RNA tunnel in the apo structure.

(C) Stacking of Tyr32 against the guanine base of G2 and binding of Lys79 and Lys204 to the phosphate backbone of the RNA creates an open RNA binding tunnel, as shown in the RNP structure.

(D) ITC binding curve of the 9mer RNA oligo1 to the following N mutants: Y32A (K_d_ = 1.6 µM ± 0.2 μM, n = 0.46 ± 0.01); K79A (K_d_ = 220 nM ± 30 nM, n= 0.42 ± 0.01); Y32A-K79A (K_d_ = 6 µM ± 2 μM, n = 0.43 ± 0.05); K204A (K_d_ = 250 nM ± 10 nM, n = 0.41 ± 0.01).

(E) EM microscopy of reconstituted RNP particles of the Toscana virus Y32A, K79A, Y32A-K79A and K204A mutants, as described in Figure 1C.

(F) Minireplicon assay of RVFV with the indicated N mutants. Cells were transfected with constructs for RVFV L, a model minigenome expressing a reporter gene, RVFV N, and the indicated N mutants. The reporter values of the minireplicon system reflect the activity of the recombinant RNPs reconstituted from expressed cDNA sequences.

**Figure 4: RNA binding induces an inter-subunit shift**

(A) Side-view of three subunits of the apo N ring showing a planar assembly of the subunits.

(B) Same view on the RNP, detailing the staggered assembly induced by RNA binding. The two peripheral subunits are displaced relative to the central subunit (indicated by arrows).

(C) One monomer of the apo structure (brown) was superimposed with a monomer of the RNP structure (white; this subunit did not contain 9mer RNA) at the 5' RNA end. The relative position of the neighboring subunits is depicted (yellow: apo structure, blue: 9mer RNA bound subunit). A magnification of the box is shown at the right. While Tyr32 of the yellow and blue monomer occupy a similar position relative to the white/brown monomer, Lys204 undergoes an 11 Å displacement.

(D) Side view of (A**)** showing that RNA binding induces a 45° tilt between neighboring subunits.

(E) View on the apo structure from the outside of the hexamer. The inter-subunit contact is almost exclusively mediated by the amino terminal arm. Arg213 stabilizes the amino-terminal arm by binding to the amide backbone of Asn28 in the same chain.

(F) Same view of the Toscana virus RNP structure showing that a new inter-subunit interface is formed upon RNA binding. Arg213 forms a hydrogen bond to the peptide backbone of Leu131 while Arg217 forms a hydrogen bond to Thr137. A new salt bridge is formed between Asp173 and Arg191 of the neighboring subunit.

(G) Minireplicon assay with the indicated mutants, as in Fig. 3F.

**Figure 5: Model for the helical assembly of Toscana virus RNP**

(A) The helical assembly of Toscana virus RNP was modeled by extending the observed inter-subunit shift at the 5' RNA end to the adjacent subunits. This transformation results in a left-handed helical filament with an external diameter of 10 nm. RNA can be bound in a continuous RNA binding groove at the inside of the helix (indicated in red). (B) shows a top view of this helix. (C) Schematic representation of the proposed steps involved in the formation of the RNP filament. Binding of longer RNA sequences to the N hexamer results in an upward shift of the neighboring subunit akin to a helical RNP. The consecutive recruitment of further subunits through both the amino-terminal arm and an outer interface formed by D173-R191 salt bridge may extend this hexamer.

**Table 1: RNA sequences used in the study and binding parameters to Toscana Virus N**

| **Oligo** | **Sequence** | **Binding affinity** | **Stoichiometry** |
| --- | --- | --- | --- |
| oligo1 (used for crystallization) | UGUGUUUCU | 260 nM ± 20 nM | 0.49 ± 0.01 |
| oligo2 (3' genomic consensus sequence) | UCUUUGUGU | 560 nM ± 70 nM | 0.43 ± 0.01 |
| oligo3 (5' genomic consensus sequence) | ACACAGAGA | n.d | n.d. |
| oligo4 (G2A mutant of oligo1) | UAUGUUUCU | 290 nM ± 40 nM | 0.49 ± 0.01 |
| oligo5 (polyU 9mer) | UUUUUUUUU | 360 nM ± 40 nM | 0.40 ± 0.01 |
| oligo6 (8mer RNA derived from oligo1) | UGUGUUUC | 710 nM ± 30 nM | 0.51 ± 0.01 |
| oligo7 (7mer RNA derived from oligo1) | UGUGUUU | 2.7 µM ± 0.2 μM | 0.52 ± 0.01 |
| oligo8 (6mer RNA derived from oligo1) | UGUGUU | 8.0 µM ± 0.9 μM | 0.50 ± 0.03 |
| Y32A + oligo1 | UGUGUUUCU | 1.6 µM ± 0.2 μM | 0.46 ± 0.01 |
| K79A + oligo 1 | UGUGUUUCU | 220 nM ± 30 nM | 0.42 ± 0.01 |
| Y32A-K79A + oligo 1 | UGUGUUUCU | 6 µM ± 2 μM | 0.43 ± 0.05 |
| K204A + oligo1 | UGUGUUUCU | 250 nM ± 10 nM | 0.41 ± 0.01 |
| RVFV N +oligo 1 | UGUGUUUCU | 400 nM ± 100 nM | 0.70 ± 0.02 |

**Table 2:** Data collection and refinement statistics

|  | Apo Nucleoprotein | RNP |
| --- | --- | --- |
| **Data collection** |  |  |
| Space group | P6, 12 protein molecules / asymmetric unit (ASU) | P1, 24 protein and 12 RNA molecule / ASU |
| Cell dimensions  *a*, *b*, *c* (Å)  *α*, *β*, *γ* (°) | 104.5, 104.5, 510.8  90, 90, 120 | 98.8, 127.8, 170.5  82.1, 79.7, 74.5 |
| Wavelength (Å) | 0.91841 | 0.91841 |
| Resolution (Å) | 100-3.32 (3.41-3.32)^a^ | 34.4-2.6 (2.61-2.59) |
| Number of observed reflections | 193,157 (11,635) | 514,757 (67,736) |
| Number of unique reflections | 45,886 (3,257)^b^ | 229,780 (31,175) |
| *R*_sym_ (%)^b^ | 12.4 (50.8) | 9.2 (55.5) |
| I/σI * | 10.7 (2.4) | 11.2 (2.1) |
| Completeness (%)* | 99.1 (94.5) | 94.7 (79.7) |
| Redundancy | 4.2 (3.6) | 2.2 (2.2) |
| ASU content | 2×(N_6_) | 4×(N_6_-RNA_(3x9mer)_) |
|  |  |  |
| **Refinement** |  |  |
| Resolution (Å) | 34.1-3.32 (3.39-3.32) | 34.4-2.6 (2.63-2.60) |
| No. reflections | 45,879 (2,741) | 229,737 (4788) |
| *R*_work_ / *R*_free_ (%)^c^ | 24.7/27.5 (34.7/36.9) | 22.5/25.8 (31.2/37.6) |
| No. of protein atoms | 22,846 | 45,778 |
| averaged B-factor protein (Å^2^) | 73.1 | 46.8 |
| averaged B-factor RNA (Å^2^) | NA | 44.3 |
| R.m.s deviations  bond lengths (Å)  bond angles (º) | 0.002  0.56 | 0.004  0.840 |
| Residues in favored region of the Ramachandran plot^d^ (%) | 95.3 | 97.3 |

^a^ Values given in parentheses refer to reflections in the outer resolution shell

^b^ R_sym_ = Σ | I(k) - <I> | / Σ I(k) , where I(k) and <I> represent the scaled intensity values of the individual measurements and the corresponding mean values.

^c^ For calculation of R_free_, 5% of all reflections were omitted from refinement.

^d^ as analyzed by Molprobity
